# Clinical Applications of Artificial Intelligence in Corneal Diseases

**DOI:** 10.3390/vision9030071

**Published:** 2025-08-18

**Authors:** Omar Nusair, Hassan Asadigandomani, Hossein Farrokhpour, Fatemeh Moosaie, Zahra Bibak-Bejandi, Alireza Razavi, Kimia Daneshvar, Mohammad Soleimani

**Affiliations:** 1 Kittner Eye Center, Department of Ophthalmology, University of North Carolina, Chapel Hill, NC 27517, USA; 2 Department of Ophthalmology, University of California, San Francisco, CA 94143, USA; 3 Translational Ophthalmology Research Center, Farabi Eye Hospital, Tehran University of Medical Sciences, Tehran 1336616351, Iran; 4 Universal Scientific Education and Research Network (USERN), Tehran University of Medical Sciences, Tehran 1416634793, Iran; 5 Department of Ophthalmology and Visual Sciences, University of Illinois at Chicago, Chicago, IL 60612, USA; 6 Eye Research Center, Farabi Eye Hospital, Tehran University of Medical Sciences, Tehran 1336616351, Iran

**Keywords:** artificial intelligence, deep learning, machine learning, corneal diseases, keratoconus, infectious keratitis, dry eye disease, tear film, corneal neuropathy, conjunctiva

## Abstract

We evaluated the clinical applications of artificial intelligence models in diagnosing corneal diseases, highlighting their performance metrics and clinical potential. A systematic search was conducted for several disease categories: keratoconus (KC), Fuch’s endothelial corneal dystrophy (FECD), infectious keratitis (IK), corneal neuropathy, dry eye disease (DED), and conjunctival diseases. Metrics such as sensitivity, specificity, accuracy, and area under the curve (AUC) were extracted. Across the diseases, convolutional neural networks and other deep learning models frequently achieved or exceeded established diagnostic benchmarks (AUC > 0.90; sensitivity/specificity > 0.85–0.90), with a particularly strong performance for KC and FECD when trained on consistent imaging modalities such as anterior segment optical coherence tomography (AS-OCT). Models for IK and conjunctival diseases showed promise but faced challenges in heterogeneous image quality and limited objective training criteria. DED and tear film models benefited from multimodal data yet lacked direct comparisons with expert clinicians. Despite high diagnostic precision, challenges from heterogeneous data, a lack of standardization in disease definitions, imaging acquisition, and model training remain. The broad implementation of artificial intelligence must address these limitations to improve eye care equity.

## 1. Introduction

Ocular diseases impose a substantial burden of blindness and visual impairment worldwide [1]. Despite the progress in lowering age-adjusted blindness since 1990, population growth, aging, and evolving lifestyles have caused a notable increase in the number of blindness and vision impairment cases. The overwhelming of ophthalmic services has been noted as a major factor behind this rise, marking the need for more efficient methods [2].

Corneal diseases, in particular, are a major contributor to the global burden of eye diseases [3]. Additionally, disorders including dry eye disease (DED), corneal neuropathy, keratoconus (KC), and infectious keratitis (IK) have substantial effects on patients’ quality of life and drive substantial economic costs [2,4,5,6,7,8,9,10,11].

There are a variety of diagnostic methods available for each of these disorders, including slit lamp examinations, various imaging devices such as anterior segment optical coherence tomography (AS-OCT), tear film assessments, in vivo corneal confocal microscopy (IVCM), and microbial cultures [12,13,14,15,16]. However, they are often time-consuming and labor-intensive, requiring precise manual analysis and limiting clinical workflow. Moreover, these approaches also demand trained specialists that are in limited supply, especially in low-resource regions.

The applications and performance of artificial intelligence (AI) have emerged as a promising tool for improving diagnostic dilemmas and addressing the current limitations. Machine learning (ML), a subset of AI, and deep learning (DL), a subset of ML, offer novel and efficient insights. They have been used for enhancing the diagnosis of multiple eye diseases [17,18]. Thus far, various algorithms and models have been developed using existing data, enabling automated segmentation, feature extraction, and predictive analytics on par with highly trained specialists [19,20,21,22,23,24,25,26].

With the continuous advancements in AI technologies, including large language models (LLMs) such as ChatGPT-4.0, becoming more accessible, their integration into clinical practice necessitates ongoing evaluation. While previous studies have explored the applications of AI in corneal disease, many focus on only one or a few corneal conditions. Our study offers a comprehensive overview of several corneal diseases and AI models, allowing for the recognition of patterns between models and diseases. Additionally, we identify the strengths and areas of improvement for this rapidly evolving field.

## 2. Methods

In this narrative study, a systematic literature search across three databases, using PubMed, Web of Science, and SCOPUS, was performed. All articles published until April 2025 were included in the search. The search strategy utilized a combination of keywords and controlled vocabulary (e.g., MeSH terms) such as “artificial intelligence,” “machine learning,” “deep learning,” “corneal diseases,” “keratoconus,” “infectious keratitis,” “corneal neuropathy,” “dry eye disease,” “corneal dystrophy,” “corneal degeneration,” “ocular surface tumors,” “pterygium,” and “conjunctival diseases,” tailored to each database’s syntax. Abstracts meeting the criteria were selected for full-text review. Two independent reviewers then assessed the full texts for eligibility. Discrepancies between the reviewers were resolved through discussion to reach a consensus on inclusion. Studies were included if they reported LLM-, ML-, or DL-based approaches (e.g., neural networks, support vector machines) applied to corneal disease diagnosis or monitoring. No language or publication date restrictions were applied initially, though primarily English-language articles were ultimately reviewed. Data extraction focused on performance metrics (sensitivity, specificity, accuracy, area under the curve [AUC]), imaging techniques, AI model types, and study limitations. Qualitative synthesis was employed to summarize the findings, with an emphasis on model performance, challenges, and clinical implications. Study quality was not formally assessed, aligning with the narrative review framework, but key trends and discrepancies were critically evaluated based on the extracted data. The study adhered to the tenets of the Declaration of Helsinki.

## 3. Results

### 3.1. Corneal Structural Diseases

#### 3.1.1. Keratoconus

Keratoconus is a progressive condition where the cornea bulges into a cone shape due to stromal thinning [27,28]. The early detection of KC is crucial for preventing unpredictable outcomes in refractive surgery and contact lens fitting [29,30,31,32]. However, identifying subclinical KC (SKC) remains a significant challenge before refractive surgery due to the overlap in topography and tomography imaging parameters between SKC and normal eyes [33,34,35,36]. The condition’s cause is multifactorial, involving genetic predisposition, eye rubbing, and biomechanical factors. A cascade of biomechanical decompensation triggered by a focal change in corneal elasticity is thought to drive KC progression [37,38,39,40,41]. ML algorithms and neural networks have the potential to determine disease progression in an unbiased manner [37,42,43,44,45,46,47,48,49,50,51,52,53,54,55,56,57,58,59,60]. For instance, DL of optical coherence tomography (OCT) color-coded maps have shown strong potential in identifying progressive from non-progressive KC with an 85% accuracy using the adjusted age algorithm [61].

Numerous studies have tested a variety of AI models in the context of KC diagnosis, screening, and prediction [61,62,63,64,65,66,67,68,69,70,71,72,73,74,75,76,77,78,79,80,81,82,83,84,85,86,87,88,89,90,91,92,93,94,95,96,97,98,99,100,101,102,103,104,105,106,107,108,109,110,111,112,113,114,115,116,117,118,119,120,121,122,123,124,125,126,127,128,129,130,131,132,133,134,135]. Most commonly, traditional ML models are utilized such as neural networks (NNs), decision trees, random forest (RF) models, support vector machines (SVMs) and more. However, DL models such as convolutional neural networks (CNNs) and generative adversarial networks (GAN) are also used. Models are trained with extensive data from corneal imaging including Placido topography, Scheimpflug tomography, slit scanning systems, OCT, and Pentacam topography. Pentacam topography, especially, has made the most significant contribution to enhancing sensitivity and specificity in ML algorithms (27 studies) [31,32,43,45,46,62,63,64,65,66,67,68,69,70,71,72,73,74,75,76,77,78,79,80,81,82,83].

Neural networks, including multi-layer perceptrons (MLPs), have demonstrated high diagnostic accuracy, with some analyses reporting a sensitivity up to 100% [84]. Our review showed that 30 studies used neural networks for KC detection [27,28,29,51,85,86,87,88,89,90,91,92,93,94,95,96,97,98,99,100,101,102,103,104,105,106,107,108,109], with most relying on corneal topography. The highest sensitivity and specificity (both 100%) were achieved in the study by Fisher et al. [94]. The FPA-K-means unsupervised algorithm, free from pre-labeling bias, also efficiently identified KC [110]. Additionally, an SVM model using eight key corneal parameters from OCT-based topography achieved 94% accuracy [111].

Random forest classifiers achieved 98% and 95% accuracy in identifying class 2 and class 4 keratoconus, respectively, using the Harvard Dataverse KC dataset with sequential forward selection [112]. Logistic regression and SVM models helped develop indices such as the Fourier-based keratoconus detection index (FKI), which demonstrated strong subclinical KC diagnostic performance [113,114,115,116,117]. SVMs using a cubic kernel and elevation parameters achieved 96.6% accuracy in distinguishing KC from normal cases [66] and reported over 93% prediction accuracy and 95% overall accuracy in other studies [32].

Naïve Bayes (NB) classifiers and clustering methods like hierarchical clustering and k-nearest neighbor were also used to stratify patient subgroups and enhance diagnostic precision [114,118]. Among various AI models proposed for KC screening, an ensemble model using soft voting achieved the highest sensitivity—90.5% on internal and 96.4% on external validation data [119]. Newer AI-based indices such as BESTi showed improved sensitivity and specificity (84.97%) for detecting SKC compared with the Pentacam Random Forest Index (PRFI) and Belin–Ambrósio Deviation Index (BAD-D) [120].

In a diagnostic study evaluating AI’s power in distinguishing non-KC, SKC, and KC cases, the global diagnostic accuracy reached 95.53%; the aforementioned model exceeded ophthalmology residents’ accuracy of 93.55%, with a validation AUC of 0.99 [121]. Automated tree-based ML classifiers reached 100% sensitivity and 99.5% specificity in differentiating KC from normal corneas [82]. Deep learning models, particularly CNNs, were trained on Scheimpflug tomography and OCT images. These DL models demonstrated excellent performance in differentiating non-KC, SKC, and manifest KC [84,115,122]. Summary sensitivity and specificity for manifest KC diagnosis reached 98.6% and 98.3%, respectively [113], while SKC detection reported a slightly lower sensitivity (90.0%) [113]. Combining CNNs with color-coded Scheimpflug images showed that the model was dependent on specific spatial regions to differentiate between KC, SKC and non-KC eyes (accuracies ranging from 0.98 to 0.99) [123]. One study cited that a combination of corneal elevation data and tomography improved SKC discrimination from normal eyes with 92.5% sensitivity and 92% specificity [124].

Other DL approaches included GANs used for data augmentation and representation learning [122]. Deep learning on OCT color-coded maps demonstrated 85% accuracy in distinguishing progressive from non-progressive KC using an adjusted age algorithm [61]. The Ectasia Status Index (ESI), developed using unsupervised ML from OCT data, effectively assessed KC severity [35]. Posterior corneal surface characteristics and thickness were also predictive of disease progression. Neural networks achieved the highest prediction accuracy (98.29%), followed by SVMs (97.72%), though SVMs performed better in terms of training and testing time [107].

In a novel approach, researchers assessed KC severity using the RETICS scale. The model achieved 85% sensitivity on validation and 95% on training data [60]. Influential risk factors included gender, coma-like aberrations, central thickness, higher-order aberrations, and temporal corneal thinning. This RETICS-based web application enables rapid, objective, and quantitative KC evaluation, aiding early diagnosis and disease grading.

Across 25 ML models that utilized OCT-based topography and multiple corneal parameters (topography, elevation, pachymetry), the most accurate model was an SVM algorithm using eight corneal features, which achieved 94% accuracy [110]. These models show strong potential for integration into clinical imaging devices or as diagnostic software for early KC detection and assessment. The keratoconus AI models are summarized in Table 1.

#### 3.1.2. Fuch’s Endothelial Corneal Dystrophy

Fuch’s endothelial corneal dystrophy (FECD) is a progressive disease characterized by corneal endothelial loss [136]. As a consequence, this leads to stromal edema and guttata formation [136]. This sequence of events can lead to significant visual impairment. Artificial intelligence has shown significant promise in the diagnosis and management of FECD, particularly through imaging modalities such as AS-OCT, specular microscopy, and slit lamp photography.

Several DL models have been developed to differentiate between early- and late-stage FECD, as well as to distinguish FECD from non-FECD corneas. Eleiwa et al., using high-definition OCT images, developed a model capable of differentiating non-FECD corneas from early and late FECD with a sensitivity of 99% and specificity of 98% [137]. The same authors using AS-OCT achieved a high accuracy in distinguishing between early-FECD, late-FECD, and non-FECD eyes. Early-stage FECD reached an AUC of 0.997 (sensitivity 91%, specificity 97%), late-stage FECD had an AUC of 0.974 (sensitivity up to 100%, specificity 92%), and distinguishing all FECD cases from non-FECD eyes achieved an AUC of 0.998 (sensitivity 99%, specificity 98%) [137]. Another AS-OCT-based DL model diagnosing DED, KC, and FECD demonstrated excellent performance, achieving AUCs of 1.0 (F1 score 100%) for FECD, 0.99 for KC (F1 98%), and 0.99 for DED (F1 90%) [138].

In studies using slit lamp photographs (SLP), Gu et al. reported an AUC of 0.939 in detecting corneal dystrophy and degeneration, including FECD, alongside other ocular surface conditions [139]. The hierarchical DL model achieved AUCs ranging from 0.903 to 0.951 in retrospective testing and >0.91 in a prospective cohort of 510 cases [139]. Another study applying semantic segmentation to SLP images reported a high diagnostic accuracy across ten anterior segment pathologies, with accuracy ranging from 79 to 99%, sensitivity from 53 to 99%, and specificity from 85 to 99% [140].

AI has also been used for the quantitative assessment of the corneal endothelium using specular microscopy [141]. DL models based on CNN U-net architectures have enhanced the speed and accuracy of morphologic analyses compared with manual assessments and conventional software [141]. In one comparative analysis, CNN-based models could estimate endothelial parameters in 98% of images with a percentage error of 2.5–5.7%, while conventional specular microscopy could only analyze 31–72% of images with higher error margins of 7.5–18.3% [142]. Despite these advancements, specular microscopy is limited in advanced FECD due to corneal edema that obscures imaging [143,144].

To overcome these limitations, salience map (SM) imaging has been explored. One study using 775 SM images reported a strong performance in internal validation (AUC 0.92, sensitivity 0.86, specificity 0.86) and a moderate performance in external validation (AUC 0.82, sensitivity 0.74, specificity 0.74) for detecting abnormal images [145]. For distinguishing FECD from other diagnoses, internal validation showed an AUC of 0.96 (sensitivity 0.91, specificity 0.91), whereas external validation showed a lower performance (AUC 0.77, sensitivity 0.69, specificity 0.68) [145]. A separate DL model identified widefield SM images with an endothelial cell density (ECD) > 1000 cells/mm^2^, diagnosing FECD eyes with a sensitivity of 0.79, specificity of 0.78, and an AUC of 0.88 [145].

A support vector machine (SVM) model was used to differentiate non-pathological and pathological corneas based on pupil size and principal components (PCs) [146]. For a 3 mm pupil analysis with three PCs, the model achieved an accuracy of 92.8%, a sensitivity of 96.9%, and a precision of 94.8%; with five PCs, the accuracy remained at 92.8%, the sensitivity was 96.1%, and the precision was 95.2% [146]. At 5 mm pupil size, performance slightly decreased but remained strong, with an accuracy around 90.2–90.7%, sensitivity of 94.8%, and precision of 92.1–92.7% [146].

Another innovation involved the use of edema fraction (EF), the ratio of pixels labeled as edema to the total corneal pixels, as a marker for early disease detection [147]. For detecting a 20 µm change in differential central corneal thickness (DCCT), EF achieved an AUC of 0.97 overall, 0.96 in FECD and normal eyes, and 0.99 in non-FECD and normal eyes [147].

Additionally, one study highlighted the importance of guttae area ratio (GAR%) as a quantitative imaging biomarker that aligns well with the m-Krachmer grading scale. However, this study was focused on the central corneal region [148].

Despite all these advances, a systemic review by Liu et al. found no studies utilizing Scheimpflug imaging (e.g., from Pentacam) for AI training in FECD [149]. They hypothesized that this was due to limitations in data accessibility from proprietary formats [149]. AS-OCT remains the preferred modality due to its superior resolution and capacity to differentiate between epithelial and stromal edema—critical for evaluating disease severity and treatment response [149].

Nevertheless, AI applications in FECD diagnosis have demonstrated excellent diagnostic accuracy, sensitivity, and specificity across imaging modalities, highlighting its potential to augment clinical evaluation, especially in early or ambiguous cases. A summary of the models discussed in this section is found in Table 2.

### 3.2. Dry Eye Disease

Dry eye disease is a prevalent eye condition worldwide, affecting between 5 and 50% of the population, depending on the diagnostic criteria and study population. DED is a multifactorial condition classified into aqueous-deficient and evaporative DED, associated with insufficient tear production and dysfunctional meibomian glands, respectively [150].

The subjective nature of diagnostic tools has made DED diagnosis challenging [151]. Key clinical signs include decreased tear volume, rapid tear film break-up, and ocular surface microwounds. However, diagnostic tests may not always correlate with symptom severity. A comprehensive DED evaluation utilizes a range of tests to assess tear film parameters, including tear film break-up time (TBUT), Schirmer’s test, tear osmolarity, and tear meniscus height [152]. Additional diagnostic tools include ocular surface staining, corneal sensibility, interblink frequency, corneal topography, interferometry, aberrometry, and advanced imaging techniques like meibography and corneal confocal microscopy (CCM) [152].

Applying AI to the analysis of DED testing and protocol development for diagnosis and disease monitoring potentially enhances the accuracy and consistency of DED diagnosis. Newer studies have integrated AI with bioinformatics tools in the analysis of biofluid markers for DED. Artificial Neural Networks (ANNs), hierarchical clustering, and RF models have been utilized alongside the protein and metabolite profiling of tears for various functions: classifying disease subgroups, differentiating DED from other ocular surface diseases (OSDs), identifying risk factors, and predicting prognosis [153]. One study utilizing a nonlinear iterative partial least squares (NIPALS) algorithm followed by a multi-layer perceptron neural network (MLP NN) achieved 89.3% accuracy in classifying tear proteome profiles of non-dry eye, dry eye, and MGD-associated dry eye individuals [153]. Another study employed a multi-layer feed-forward network trained on a seven-biomarker tear panel reported an AUC of 0.93 [153]. Despite their strong performance, these models were limited in interpretability and generalizability due to their “black-box” approach [153].

Additionally, AI-driven tools have been used to analyze temperature profiles from thermal images of the cornea to distinguish between non-ADDE eyes and those with Aqueous Deficient Dry Eye (ADDE) [154]. One study using an infrared thermography (IRT) model reported 84% sensitivity, 83% specificity, and AUC of 0.87 [141]. Other IRT studies using ML classifiers like PNN, KNN, and SVM reported near-perfect metrics, with one achieving 99.8% sensitivity, specificity, and accuracy (left eye), and another reaching 99.9% sensitivity, 99.4% specificity, and 99.8% accuracy (right eye) [154]. A KNN-based model with ten-fold cross-validation reported 99.88% accuracy, 99.7% sensitivity, and 100% specificity [154].

Cartes et al. found that the tear film osmolarity variability was higher in individuals with dry eye syndrome compared with non-dry eye controls. They created a classification model with an initial accuracy of around 85%, emphasizing the potential diagnostic value of tear osmolarity in distinguishing between non-DED individuals and those with DED [155].

The analysis of tear proteins’ electrophoretic patterns using an ML-based ANN achieved an accuracy rate of 89% in diagnosing DED. The ANN demonstrated the ability to detect signs of DED from electrophoretic patterns [156].

Protein chip array profiling of tear samples, combined with AI analysis, achieved 90% sensitivity and specificity in distinguishing DED patients from non-DED controls [157].

The creation of innovative diagnostic algorithms, based on TBUT, is a significant advancement in the assessment of DED as well. Combining video-based TBUT analysis with advanced computational techniques demonstrates the value of using new technologies to tackle challenges linked to diagnosing and grading DED. In 2007, Yedidya et al. developed an algorithm using the EyeScan system to automatically detect DED by analyzing slit lamp videos for the first time. Their initial method achieved 91% accuracy compared with optometrist assessment and was later expanded to include additional tear film metrics [158]. Su et al. conducted a study to explore the potential of CNN technology in evaluating tear film stability by analyzing fluorescein TBUT (FTBUT). They developed a CNN-based method to segment fluorescent images and detect areas of broken tear film using a 5 s cutoff value after blinking. The method achieved a high accuracy rate of 98.3% in detecting the break-up area, highlighting the effectiveness of CNN-based FTBUT assessment as a reliable tool for evaluating tear film stability and screening for DED [159].

Vyas et al. proposed a new algorithm that uses TBUT data from video recordings to diagnose and grade the severity of DED. Their approach achieved 83% accuracy in detecting TBUT frames and classifying the severity of DED as normal, moderate, or severe [160].

Su et al. investigated the effectiveness of CNN classifiers in detecting superficial punctate keratitis (SPK), an important clinical feature of DED. The trained CNN classifiers achieved an accuracy of 97% in detecting punctate dots observed through fluorescein staining. Additionally, the proposed CNN-SPK grading system effectively estimated the coverage of punctate dots and demonstrated a strong correlation (r = 0.81, *p* < 0.05) with clinical gradings [161].

The utilization of tear film lipid interferometer imaging has shown promise in aiding the classification of DED [149]. Recent studies have used ML techniques to develop predictive models that analyze tear film lipid interferometer images. These models have shown a strong positive correlation with Schirmer test values and a high level of agreement (0.82) in accurately classifying the images into healthy, aqueous-deficient DED, or evaporative DED categories [162,163].

Moreover, the use of DL algorithms to study blink patterns in video recordings has shown promising results in assessing DED. Recent studies have shown the frequency of incomplete blinking, as measured by the DL-based algorithm, is closely correlated with the clinical presentation of DED [164,165].

In a more recent study, researchers used blink videos to study spontaneous blink patterns in patients with DED. They used a U-Net image segmentation algorithm to identify complete and partial blink patterns and developed a ResNet-based DL model to classify the blink videos [166]. The study found that DED patients exhibit a higher incidence of partial blinks, shorter closure time, and reduced blink amplitude compared with non-DED controls [166]. The U-Net segmentation model achieved 96.3% accuracy, and the classification model achieved 96.0% accuracy [166].

By using AS-OCT corneal epithelial mapping data, researchers created a diagnostic AI algorithm using random forest regression. This approach yielded outstanding performance results, with a sensitivity of 86.4% and a specificity of 91.7% [167]. Additionally, superior intermediate epithelial thickness was identified as a potential AS-OCT marker for diagnosing DED (AUC: 0.87) [167]. The difference between inferior and superior peripheral epithelial zones was identified as the best marker for grading DED [167].

A recent study used a rapid, non-invasive DED screening algorithm combining the Symptom Assessment in Dry Eye (SANDE) questionnaire and NIBUT, using optimal cutoffs of SANDE ≥ 30 and NIBUT < 10 s. The results exhibited 86% sensitivity and 94% specificity for detecting DED according to Tear Film and Ocular Surface Society Dry Eye Workshops (TFOS DEWS II) criteria, indicating its potential to facilitate the efficient clinical identification of the condition [168].

According to a meta-analysis of AI usage in the diagnosing of DED, the overall accuracy of AI models was found to be 91.91% (95% confidence interval: 87.46–95.49) with a sensitivity of 89.58 (±6.13) and a specificity of 92.62 (±6.61) [169]. These data indicate that the combination of bioinformatics and AI-driven technologies in the diagnosis, management, and mass screening of DED potentially improves the accuracy, consistency, and objectivity of DED assessment. The DED AI models are summarized in Table 3.

### 3.3. Tear Film

Just as a combination of biofluid markers and AI-driven tools were utilized for DED, similar methodologies have been implemented for tear film analysis. These models are guiding biomarker discovery, differentiation between various OSDs, and making prognostications in clinical settings.

Santos et al., for instance, developed a fully automated model to quantify in vivo tear film thickness using high-resolution OCT. The model demonstrated a relative accuracy of 65% and excellent reproducibility, which can aid in DED diagnosis and treatment evaluation [170].

Tear meniscus height (TMH) is a valuable diagnostic tool in the assessment of aqueous-deficient DED. Researchers have developed an automated method using a CNN to segment the tear meniscus area and calculate TMH [171]. This system achieved an average Intersection of Union (IoU) of 82.5% and demonstrated a higher correlation (0.965) with ground truth data compared with manual measurements (0.898) [171]. The improved accuracy and consistency of this approach suggests the potential to enhance the diagnosis and monitoring of aqueous-deficient DED [171].

Stegmann et al. utilized ultrahigh-resolution OCT to assess various tear film parameters, including TMH, tear meniscus area, tear meniscus depth, and tear meniscus radius. Using conventional image processing algorithms, the researchers accurately segmented the tear meniscus and quantified these crucial metrics, revealing significant correlations among them (all *p* < 0.001, all r ≥ 0.657) [172].

Moreover, they developed an ML-based approach to segment the lower tear meniscus using AS-OCT images. Their thresholding-based algorithm demonstrated exceptional performance, achieving a sensitivity of 96% and a specificity of 100% [173]. This automated segmentation technique represents a significant advancement in the objective quantification of tear meniscus parameters, which is crucial for the assessment and management of DED [173]. A summary of tear film models is shown in Table 4.

### 3.4. Infectious Keratitis

Infectious keratitis is a sight-threatening disease that requires early diagnosis due to its significant morbidity and potential for poor outcomes [174,175,176]. Traditional diagnostic tools, such as slit lamp-based evaluations, remain the cornerstone of IK diagnosis [177]. However, the visual interpretation of microbial keratitis (MK) patterns is highly subjective and requires extensive clinical experience [178]. Standard diagnostic methods like corneal scrapings and cultures are time-consuming, expertise-dependent, and often yield false-negative results due to their low sensitivity [178,179,180,181]. These limitations have driven the development and adoption of AI, particularly DL and ML, as more efficient and accurate alternatives [182,183,184,185,186].

AI models for IK classification commonly utilize images from various modalities, including slit lamp, digital anterior segment photographs (ASP), and IVCM [182,183,184,185,186,187]. These models serve several diagnostic functions: distinguishing infectious from noninfectious keratitis, differentiating among bacterial, fungal, viral, and Acanthamoeba keratitis, and even identifying fungal subtypes like yeast versus filamentous species [182,183,184,185,186,187].

As early as 2003, Jagjit S. Saini et al. introduced neural networks for identifying bacterial keratitis (BK) and fungal keratitis (FK), achieving an accuracy of 90.7% and outperforming clinician diagnoses [188]. Since then, AI applications have expanded significantly; CNN-based DL models developed for slit lamp photographs demonstrated a high diagnostic accuracy in multiple studies. One study developed three models: one for diagnosing IK (accuracy 99.3%), another for distinguishing BK from FK (accuracy ~84%), and a third for classifying yeast versus filamentous fungi (accuracy 77.5%) [183].

More complex DL systems, like DeepIK, mimicked expert diagnostic processes using a two-stage classifier: one to distinguish infectious from noninfectious keratitis and another to classify specific etiologies. DeepIK outperformed other DL models like DenseNet121, InceptionResNetV2, and Swin-Transformer, in line with reference standards (Cohen’s Kappa 0.70–0.77) and rapid image processing (0.034 s/image) [185]. VGG19, ResNet50, and DenseNet121 were evaluated on 2167 mixed BK and FK images; VGG19 outperformed the others with an F1 score of 0.78 and AUPRC of 0.86 [189].

Large-scale DL evaluations have further validated these models. For instance, an ensemble model analyzing 2167 slit lamp photos achieved a high performance in differentiating BK from FK, with sensitivity, F1 scores, and AUPRC of 0.77, 0.83, and 0.904, respectively [190,191]. Other models trained on 1330 cropped images reached a diagnostic accuracy of 80.0% for BK and FK [190]. MobileNet achieved the highest performance among five CNNs, with an AUC of 0.86 in single-center and 0.83 in multi-center tests [191]. ResNet-50 CNN models showed a specificity and sensitivity of 80% and 70% for BK, and 70% and 80% for FK, respectively [192].

Advanced AI methods also analyze lesion characteristics. Natural language processing (NLP) algorithms quantified microbial keratitis (MK) centrality, thinning, and depth with sensitivities ranging from 50% to 100% [193]. EfficientNet B3 achieved a sensitivity and specificity of 74 and 64, respectively, for BK diagnosis, comparable to ophthalmologists [194].

For lesion segmentation, models like SLIT-Net and multi-scale CNNs with ResNeXt achieved up to 88.96% diagnostic accuracy [195,196]. Region-based CNNs reached Dice similarity coefficients between 0.74 and 0.76 for segmenting stromal infiltrates, hypopyons, and other features [197]. ResNet50-based multi-attribute networks identified keratitis characteristics with 89.51% accuracy [198]. Ming-Tse Kuo’s DenseNet model used corneal photos for FK diagnosis, and semi-automated algorithms measuring epithelial defects (ED) showed high reliability (ICC 0.96–0.98) compared with ophthalmologists (ICC 0.84–0.88) [199,200,201].

Image enhancement techniques like histogram matching fusion (HMF) have been applied to AlexNet and VGGNet models, yielding accuracies as high as 99.95% [202]. Confocal texture analysis using an adaptive robust binary pattern (ARBP) reached 99.74% accuracy, with a TPR, TNR, and AUC near 1 [203]. A ResNet-based DL model for FK via IVCM demonstrated a high AUC (0.987), accuracy (0.962), sensitivity (0.918), and specificity (0.9834) [204].

Web-based models using confocal microscopy and networks like AlexNet, ZFNet, and VGG16 showed excellent performance, with VGG16 achieving a high accuracy (0.992), sensitivity (0.993), specificity (0.992), and AUC (0.999) [205]. Slit lamp photo datasets used by Li et al. and others covered thousands of cases. One DL model processed 1772 slit lamp photos to classify corneal disorders, while another processed 5325 images to distinguish between IK subtypes, neoplasms, and dystrophies, with AUCs over 0.910 [140].

In another study, a hybrid DL model on 4306 images yielded the following accuracy and AUC: 90.7% and 0.963 for bacteria, 95.0% and 0.975 for fungi, 97.9% and 0.995 for Acanthamoeba, and 92.3% and 0.946 for HSV, respectively [206]. A separate CNN study using 10,739 images achieved accuracies of 91.91%, 79.77%, and 81.27% for BK, FK, and Acanthamoeba, respectively [207].

Models using 928 slit photos achieved near-perfect training and testing accuracies of 100%, 99.1%, and 99.6% across AlexNet, VGG-16, and VGG-19 networks [208]. In sequential-level DL using 362 photos, diagnostic accuracies for BK, FK, and HSK surpassed 121 ophthalmologists at 78.7%, 74.23%, and 75.1%, respectively [209]. Visual Concept Mining (VCM) improved classification through pixel-level saliency analysis, with DenseNet121 yielding the best F1 scores (0.431 for BK, 0.872 for FK, 0.651 for HSK) [210]. DenseNet121 also outperformed ResNet50 and InceptionV3 in classifying healthy eyes versus BK, FK, and HSK with a 72% accuracy [211]. Additionally, HSV necrotizing stromal keratitis classification using DenseNet on 307 images showed 72% accuracy, 0.73 AUC, 69.6% sensitivity, and 76.5% specificity [212]. Other CNN models distinguished between active and scarred keratitis with 78.2% sensitivity and 91.3% specificity [213]. SVM classifier models were able to classify ulcer severity better than type, however [214].

For AK diagnosis, a CNN based on IVCM images (HRT3) differentiated AK from nonspecific findings with 76% accuracy, sensitivity, and specificity [185]. Meanwhile, ML models including logistic regression, random forest, and decision trees have also been explored for FK diagnosis, alongside lasso regression for assessing clinical signs [183].

Systematic reviews report a varied performance: a pooled accuracy of 64.38% for BK vs. FK classification and 96.6% for infectious vs. noninfectious keratitis classification [180]. DL was found to outperform human experts in all comparative studies [180]. IVCM-based DL models were more effective than ASP-based ones, with a higher sensitivity (91.8% vs. 86.2%) and specificity (94.0% vs. 83.6%), likely due to better image consistency and patient selection [181]. A summary of keratitis models can be seen in Table 5.

### 3.5. Corneal Neuropathy

Diabetes adversely impacts corneal nerves through reduced sensitivity and epithelial regeneration, thereby potentially making the corneal nerve plexus a surrogate for diabetic peripheral neuropathy [215]. Manifestations range from decreased sensitivity to vision-threatening ulcers. Changes include decreased branching, reduced sub-basal density, and increased tortuosity, potentially representing regeneration [215]. Moreover, the cornea’s integrity relies on its dense innervation, enabling the protection of its tear film through reflex arcs. The disruption of the tear film in DED leads to epithelial and nerve damage, resulting in nerve dysfunction that drives DED progression, ocular pain, and neuropathic symptoms [216]. The sub-basal plexus is more accessible for in vivo confocal evaluation than stromal nerves. The IVCM evaluation of corneal nerve fiber length (CNFL) is a valuable diagnostic tool, but current manual methods lack efficiency and objectivity. Rapid and automated segmentation techniques for evaluating the morphology of sub-basal corneal nerves are essential for the accurate diagnosis and effective management of corneal neuropathies [217].

Preston et al. designed an AI-based algorithm that could classify CCM images of patients without the need for prior image segmentation [218]. The algorithm demonstrated high levels of sensitivity, correctly identifying healthy control subjects with a sensitivity of 1.0 [218]. Furthermore, it achieved sensitivities of 0.85 in detecting patients with diabetic peripheral neuropathy (DPN) and 0.83 for patients without DPN [218].

Salahoudin et al. developed a novel, automated AI-based algorithm that utilized CCM images to rapidly quantify corneal nerve fiber length and classify DPN patients [219]. The algorithm, built upon a U-Net network and adaptive neuro-fuzzy inference system, achieved an AUC of 0.95 (92% sensitivity, 80% specificity) for detecting patients with and without DPN [219]. Furthermore, the algorithm demonstrated an AUC of 1.0 (100% sensitivity, 95% specificity) in discriminating healthy subjects from DPN patients, surpassing the ACCMetrics method [219].

Scarpa et al. developed a CNN model that utilized three non-overlapping CCM images from each eye to discriminate between healthy controls and individuals with DPN [220]. The CNN model demonstrated excellent performance, achieving a sensitivity of 0.98, a specificity of 0.96, and an overall accuracy of 0.97 in this classification task [220].

William et al. developed an innovative model that can automatically segment sub-basal nerve plexus fibers in CCM images, demonstrating promising diagnostic capabilities with a sensitivity of 0.68, a specificity of 0.87, and an AUC of 0.83 in detecting DPN [221]. Importantly, the model outperformed ACCMetrics in quantifying and evaluating corneal nerve fiber morphology [221].

Mou et al. developed a CNN-based deep grading algorithm that demonstrated a superior performance in segmenting and quantifying corneal nerve tortuosity, achieving 85.64% accuracy in four-level classification [222]. This automated DL algorithm identified significant differences in nerve tortuosity between diabetic patients and non-diabetic controls [222]. The results of these models are summarized in Table 6.

### 3.6. Conjunctiva

Ocular diseases affecting the conjunctiva, such as conjunctivitis and DED, significantly impact vision and quality of life. A wide range of AI models have been explored for the diagnosis and classification of conjunctival and ocular surface diseases, demonstrating promising results across multiple modalities and disease categories. Traditionally, conjunctival assessment relied on subjective clinician evaluations. However, the introduction of AI-powered diagnostic tools will potentially revolutionize the objectivity of conjunctival examination [223].

#### 3.6.1. Vascular Assessments

AI tools have been applied to various types of vascular assessments and disease diagnostics. For instance, Delgado-Rivera et al. applied CNN to segmented conjunctival images and achieved a 77.58% sensitivity in detecting anemia compared with laboratory test results [224].

Derakhshani et al. explored two approaches to assess conjunctival vascularity from color digital images, with the best method achieving a 0.89 correlation between predicted and actual values using an ANN [225]. Conjunctival blood velocity is known to decrease in various ocular diseases such as diabetic retinopathy and DED; hence, it serves as a valuable indicator of disease progression. However, eye movements during image acquisition and motion artifacts can hinder the accuracy of conjunctival blood flow segmentation and velocity measurement. To address this challenge, Jo et al. developed a motion correction algorithm and a segmentation approach to blurred images based on the Attention U-Net architecture [226]. Owen et al. developed an automated algorithm for measuring conjunctival vessel width, which demonstrated high session reliability and strong agreement with manual assessment methods on digital photographs [227]. Given the clinical value of monitoring ocular interventions and diseases, such as diabetes, this automated approach can facilitate the accurate and rapid assessment of conjunctival vessels [227].

Researchers developed a neural network system trained on the Japan Ocular Allergy Society (JOAS) criteria to accurately grade the severity of conjunctival hyperemia, successfully determining the vessel coverage area in 71.8% of images, with a strong correlation (r = 0.737, *p* < 0.01) between the model’s predictions and expert assessments [228].

The study by Li et al. showed that diabetes could be detected from conjunctival images with 75.1% accuracy [229]. Changes in the conjunctival microcirculation reflect diabetic vasculopathy, with 78.7% sensitivity and 69.0% specificity for type 2 diabetes diagnosis [229].

For ocular surface neovascularization, a fine-tuned U-Net model on 120 annotated slit lamp images showed excellent segmentation capabilities as well. The IoU scores for detecting total corneal area ranged from 90.0% to 95.5%, and for non-vascularized regions from 76.6% to 82.2%, with specificity values above 96% for both [230].

#### 3.6.2. Ocular Surface Tumors

A recent systemic review discussed the usage of deep learning algorithms in combination with anterior segment swept-source OCT (AS SS-OCT). Studies showed success in the detection and classification of angle closure glaucoma, with near-perfect sensitivity and specificity [231]. These models were also able to strongly estimate visual acuity in patients with senile cataracts [231]. The usage of AS SS-OCT as an imaging modality for AI models offers potential in extending its use in diagnosing conjunctival diseases such as conjunctival tumors and structural lesions for future studies.

Yoo et al. developed a CNN model to diagnose various conjunctival conditions, including rare diseases such as conjunctival melanoma, which has an incidence as low as 0.3 per 1,000,000 [232]. Given the limited image data available for training, the researchers employed data augmentation techniques to enhance the size and diversity of the training dataset. With this data augmentation approach, the CNN model achieved an accuracy of 97% in the detection of conjunctival melanoma using smartphone-captured images [232].

Other studies have demonstrated a high diagnostic accuracy for detecting ocular surface tumors using AI as well. A YOLOv5-based DL model applied to slit lamp images captured via smartphones achieved an AUC of 0.997 for ocular surface tumors, and a similarly high accuracy for corneal scars and corneal deposits [233]. The CorneAI platform, also using smartphone and slit lamp images, significantly improved ophthalmologists’ diagnostic accuracy, from 79.2% to 88.8% overall (*p* < 0.001), with specialists improving from 82.8% to 90.0% and residents from 75.6% to 86.2% [234]. CorneAI’s own accuracy was 86%, and its use enhanced physicians’ performance beyond its standalone output [234]. However, individual task performance varied: CorneAI achieved an AUC of 0.62 for tumor detection and 0.71 for deposits [235].

Other DL models such as ResNet50V2, YOLOv8x, and VGG19 trained on 2774 IVCM images, including of ocular surface squamous neoplasias (OSSNs), achieved binary classification accuracies above 97%, with precision ≥ 98%, recall ≥ 85%, and F1 scores ≥ 92% [236]. Furthermore, DL has shown potential for OSSN subtype stratification, aiding in potentially personalizing treatment plans [237].

The ocular surface pretrained model (OSPM)-enhanced classification model (OECM), trained on 1455 histologically confirmed ocular surface tumor (OST) images, demonstrated AUCs ranging from 0.891 to 0.993 across internal, external, and prospective datasets [238]. OECM significantly outperformed conventional CNNs and matched senior ophthalmologists in performance while enhancing the diagnostic capabilities of junior ophthalmologists [238].

Laslty, another study using 398 publicly available ocular surface images and CNN models such as MobileNetV2, NASNet, GoogleNet, ResNet50, and InceptionV3 were tested. MobileNetV2 performed best for conjunctival melanoma detection (AUC: 0.976, accuracy: 96.5%) [239]. When synthetic images generated via GANs were added, model performance improved further—MobileNetV2 reached an AUC of 0.983 and an accuracy of 97.2% [239].

#### 3.6.3. Pterygium

The pterygium is a fleshy conjunctival growth that extends onto the cornea and can obscure vision [240]. AI applications for pterygia span from segmentation to classification. A comprehensive review of 33 studies highlighted the evolution from manual image segmentation to end-to-end DL-based diagnosis [241]. Though DL models match ophthalmologists in accuracy, variability in clinical manifestations and the absence of a standardized grading system remain challenges [241].

Several studies underscore DL’s high performance in pterygium detection. One ensemble model trained on 172 anterior segment images achieved an accuracy of 94.12% and an AUC of 0.980 [242]. In a binary classification task involving 367 normal and 367 pterygium images, the VGG16 model achieved 99% accuracy, 98% sensitivity, 99.33% specificity, a Kappa of 0.98, and an F1 score of 99% [243].

Segmentation models also performed well. One CNN-based model using 489 slit lamp images achieved Dice coefficients of 0.9620 for cornea and 0.9020 for pterygium segmentation. The Kappa agreement with clinical visual inspection was 0.918 [244]. Another DL model distinguishing among primary, recurrent, and no pterygium in 258 eyes achieved 91.7% for sensitivity, specificity, accuracy, and an F1 score of 0.846 [245].

The RFRC (Faster RCNN + ResNet101) detection model and SRU-Net (SE-ResNeXt50-based U-Net) segmentation model, trained on 20,987 slit lamp and 1094 smartphone images, achieved 95.24% detection accuracy [246]. The fusion segmentation model achieved a microaverage F1 score of 0.8981, a sensitivity of 0.8709, a specificity of 0.9668, and an AUC of 0.9295, demonstrating robust performance across smartphone brands and matching experienced clinicians [246].

Two DL algorithms were developed to detect any pterygium and referable pterygium using anterior segment photographs. For any pterygium, the AUROCs were 99.5% (internal set, sensitivity = 98.6%, specificity = 99.0%), 99.1% (external set 1), and 99.7% (external set 2) [247]. For referable pterygium, AUROCs were 98.5% (internal), 99.7% (external set 1), and 99.0% (external set 2), confirming high sensitivity and specificity across settings [247].

In another study using 436 anterior segment images, MobileNetV2 again showed strong results with a sensitivity of 0.8370, a specificity of 0.9048, and an F1 score of 0.8250 [248].

Finally, the MOSAIC system—a multimodal assessment tool using gpt-4-turbo, claude-3-opus, and gemini-1.5-pro—demonstrated 86.96% accuracy in detecting ocular surface diseases and 66.67% accuracy in grading pterygium using 375 smartphone-acquired images [249]. This highlights the growing role of large language models in multimodal diagnostic pipelines.

Collectively, these studies showcase the diverse and high-performing AI approaches in diagnosing and managing conjunctival and ocular surface disorders, particularly emphasizing their potential as clinical decision support tools and screening instruments.

#### 3.6.4. Infectious Conjunctivitis

Similarly to applications of AI in DED and tear film, the use of molecular biomarkers in combination with AI is an area of critical interest. A recent study used ML algorithms to analyze RNA sequencing data from conjunctival samples of patients with presumed infectious conjunctivitis [250]. The primary goal was to predict corneal involvement, a marker of disease severity, based on gene expression profiles. SHAP (Shapley Additive Explanations) values identified apolipoprotein E (APOE) as a key gene associated with corneal involvement [250]. The model’s performance dropped significantly when APOE was excluded, underscoring its potential clinical value [250]. By identifying biomarkers that predict disease progression or severity, the models could eventually guide treatment decisions and prognostication in clinical settings. A summary of all conjunctival model results is in Table 7.

### 3.7. Large Language Models in Corneal Disease

LLMs are text-based AI models that have gained massive popularity over the past few years, with several new studies being published using LLMs in the diagnosis of corneal diseases. Some examples of LLMs include ChatGPT-4.0, DeepSeek V3, Qwen 2.5 MAX, Claude3.5 Sonnet, Grok3, Gemini 1.5 Flash, or other variations of these models. A few studies compared the performance of LLMs on text-based clinical case reports and vignettes against each other and expert ophthalmologists [251,252,253]. The results of the studies are summarized in Table 8. Generally, the models that performed the best were ChatGPT-4.0 and DeepSeek V3 [252,253]. Human cornea specialists, however, generally outperformed LLMs [254]. Earlier models, especially, performed the worst among all models tested, regardless of LLM type [251,252,253].

## 4. Discussion

This review provides an overview of different AI models used in diagnosing corneal diseases. We noticed a diverse range of models and imaging modalities utilized in the various studies cited. However, many of these studies did share the same performance metrics extracted during our literature search: accuracy, AUC, sensitivity, and specificity.

According to many published reporting guidelines, an AUC greater than 0.90 or sensitivity/specificity exceeding 0.85–0.90 is considered “excellent” or “clinically meaningful” for AI diagnostic standards [255,256,257]. Across all types of corneal diseases examined in this paper, AI has proven itself to meet and at times even exceed these standards. For example, a deep learning model trained on OCT topography exceeded the diagnostic accuracy of keratoconus compared with resident ophthalmologists [121]. Their ability to differentiate between subclinical KC, KC, and non-KC corneas is excellent [81,113,115,121,122,123], showing promise in future clinical applications. However, these metrics cannot be taken simply at face-value. It is important to note that these studies are retrospective [81,113,121], which possess inherent biases especially in patient selection for case–controlled studies. Additionally, there was a high degree of variability in the variables and imaging modalities the models were trained on.

Studies evaluating the performance of FECD demonstrated high performance metrics, often meeting or exceeding reporting guidelines [137,138,139,140,142,145,146,147]. Models trained for FECD evaluation used a variety of different imaging sources for training. Most commonly, AS-OCT, which is also deemed the most preferred form of imaging [137,138,149]. Other modalities such as SLP and specular microscopy were used. Some studies creatively implemented salience maps, edema fraction, and guttae area ratio as model variables as well [139,140,141,142,145,147,148]. While some models had objective variables such as the Krachmer scale or edema fraction to compare between eyes [145,146,147], others did not [138,139]. Feeding the models images for training purposes without objective metrics for analysis contributes to the “black box” approach in their decision-making capabilities.

Several models for dry eye disease and tear film assessment achieved high diagnostic metrics as well [153,154,155,156,157,158,159,160,161,162,163,164,165,166,169,171,172,173]. Many of these models integrated clinical signs, biomarkers, and imaging to improve objectivity and accuracy. Unfortunately, due to the fact that data providing direct comparisons to expert clinicians was limited, it affects their clinical adoption. Additionally, there was great variability in the number of images used to train these models, ranging from double digits to thousands. Some studies tried to mitigate the limited sample size by implementing multiple training cycles [155]. Others did not specify the quantity of images used, which should warrant caution in interpreting their results for generalizability and reproducibility [154,166,170].

In the diagnosis of infectious keratitis, several AI models have been used to differentiate between types of keratitis, even as specific as yeast versus filamentous fungi [182,183,184,185,186]. DeepIK matched Cohen’s Kappa reference standards, indicating agreement with expert-level diagnosis [181]. Similarly, ensemble CNNs reached F1 and AUPRC metric values that indicate deep learning models can achieve robust results even in imbalanced classification settings [189,190]. Just as there were many models without objective criteria to screen for in FECD training images, the same applies for IK models [183,185,190,191,192,194,199]. Nevertheless, some models did use specific variables and annotation methods to train their models [188,189,193,200,201,202,203,204,205]. However, AI models for IK still face challenges such as image quality variability and risk of overfitting [180]. To improve generalizability, future research should incorporate multivariate models that combine clinical history, physical exam findings, and imaging data [184].

For conjunctival disease, a neural network trained on JOAS criteria exhibited strong agreement with expert grading (r = 0.737, *p* < 0.01), suggesting that AI can match clinician assessments in more subjective domains as well [228]. Even more impressive is the ability to diagnose ocular surface tumors, such as conjunctival melanoma, with simple smartphone images to such a high degree of accuracy [232,233,234,235]. The same can be seen with models trained to classify pterygia [242,243,244,245,246,247] and nerve morphologies for corneal neuropathy [219,220]. The main limitation for studies in this category of disease were the high variability in image quality and quantity [224,225,226,227,228,229,232,233,234,235,236,237,238,239,240,241,242,243,244,245,246,247,248,249,250]. Some studies with smaller sample sizes utilized a multi-fold cross-validation technique to account for this limitation as well [232,242].

Large language models have become popular in recent years, with significant improvements in diagnostic capabilities over the past few generations of models. These models have been tested with diverse sets of corneal cases and vignettes. The best performing LLMs were the newer generation ChatGPT and DeepSeek models [251,252,253]. Despite these advancements, they do not quite measure up to the “excellent” standards set by AI diagnostic guidelines. They still do perform well overall, however, with studies citing their potential utility in augmenting clinical decision-making [251,252,253,254]. More studies using LLMs are still emerging and warrant further exploration. There are currently a limited number of studies on the use of LLMs in corneal disease, far too limited to make any broad generalizations at this time.

Collectively, these findings across all types of ocular surface diseases show that AI models are able to achieve a high diagnostic accuracy aligning closely with expert-level performance, or at least enough to consider augmenting clinical practice. Despite the fact that many models performed highly in this review, certain models outshined others in comparison. Across all types of corneal disease, neural networks performed well, specifically, CNNs consistently performed the best compared with other types of models [107,115,122,123,159,161,166,181,184,205,206,209,220,222,232,239,244,246]. Deep learning models typically outperformed traditional machine learning, large language, and algorithm-based models. Deep learning models especially outperformed traditional machine learning and algorithm-based models in image analysis and expert-level classification tasks [107,115,121,122,123,137,138,139,140,141,146,159,161,166,181,184,205,206,209,220,222,236,237,238,239]. Several ML models performed excellently in dry eye disease and tear film analysis [152,154,156,157,167,172,173]. Meanwhile, algorithm-based models excelled in screening tasks, such as the thresholding segmentation of tear meniscus and fixed-cutoff screening tools (SANDE + NIBUT) [168,173]. Lastly, LLMs performed well in text-based clinical cases, offering potential for augmenting clinical practice, though not comparable enough to experienced ophthalmologists [251,252,253,254].

While different AI models were created or trained differently by the investigators, we noticed a lack of standardization in model development and reporting. Even with similar models, there are inconsistencies in the cohort size, imaging modality used, methods for result validation, and ratio for dataset splitting to train the models [113,117,122,136,137,138,141,148,152,183,185,258]. While it may be difficult to standardize AI models due to a multitude of variables, such as what is being studied and institutional-, disease-, or patient-specific limitations, we recommend the standardization of reporting information in AI studies. In fact, standardization tools for AI studies already do exist, such as the STARD-AI [257] and QUADAS-AI [259] checklists. The STARD-AI checklist requires the clear reporting of study design, methodology, and evaluation to ensure transparency in AI diagnostic research. Meanwhile, QUADAS-AI assesses bias risk and methodology quality. These tools should be applied when evaluating these studies, especially in systemic reviews and meta-analyses, to allow fair comparisons between model types. Some systematic reviews in our review did apply the QUADAS-2 checklist [84,115,122,149,181,182]. Yet quite a few studies did not or utilized assessment tools not specific to AI studies [117,132,133,151,169,231,258].

Without the consistent application of these tools, it becomes challenging to evaluate AI models fairly or implement them in the clinical setting. Nevertheless, the successful integration of AI into healthcare systems could alleviate healthcare burden and reduce health disparities, especially in low-resource or -access settings [260]. For example, the Ophthalmologist Robot was developed to automate the collection of ocular surface and fundus images [261]. This robot supports the early detection of sight-threatening disease through eye screening and AI integration [261]. Innovations such as this one, along with the AI models evaluated in this review, could enable earlier interventions and reduce healthcare burden, though further studies are needed to confirm their impact on health equity.

Studies implementing smartphone images and smartphone-based AI tools offer a promising solution to eye care access in underserved areas as well [232,233,234,235,246,249]. These new technologies may advance the field of teleophthalmology. However, despite their auspicious results, the performance in real-world scenarios rather than a controlled environment may yield different results. Hence, future studies should take these tools a step further by focusing on user training, validation using different smartphone devices, and diversifying the datasets.

In the realm of healthcare access, ML models have also been effective in triaging patients with an elevated risk of developing ocular pathologies. For instance, one model achieved an AUC of 0.803 for ocular surface diseases requiring medication [262]. These algorithms could empower primary care providers to identify patients who would otherwise go unnoticed [262]. Therefore, AI integrations hold potential in allowing timely referrals that mitigate disease progression.

However, the integration of AI into ophthalmology necessitates an emphasis on ethical considerations. Transparency in model development, validation using diverse data, and performance monitoring can minimize the risk of bias and data misuse. Ethical oversight using tools such as the STARD-AI and QUADAS-AI could prevent the propagation of existing healthcare inequities, especially among socioeconomically disadvantaged populations.

Looking forward, the evolution of AI in ophthalmology must be guided by the technical expertise of AI developers, ophthalmologist clinical insights, and a universal ethical foundation. As AI advancements in ophthalmology continue, its success will be judged by its ability to equitably transform care delivery and reduce preventable blindness.

## 5. Conclusions

Several AI models have been developed for diagnosing corneal diseases. Many of these models perform well according to published practice guidelines. Deep learning models specifically seem to outperform other models; however, other models also perform strongly in their defined tasks. AI will have a potentially profound impact on maximizing diagnostic efficiency and providing equitable care in under-resourced regions. However, with the rise in AI technologies, it is prudent to consider standardizing model comparison metrics, diagnostic definitions, and training methodologies. Standardization tools such as the STARD-AI and QUADAS-AI checklists already exist and would allow just comparison between these diverse model sets. Furthermore, these tools, along with consistent experimental methodologies, will ensure the ethical oversight of newly developed models.

## Figures and Tables

**Table 1:** Summary of keratoconus models.

AI Model	Imaging Modality	Performance Metrics	Notes
Random Forest [112]	Pentacam, Harvard Dataverse (topography); 434 eyes	Accuracy: 98%, 95%	Used sequential forward selection (SFS) for feature selection
Support Vector Machine [32,66,111]	3151 OCT images [113], Pentacam parameters from 5881 eyes [67] and 3502 eyes [32] (elevation and topography)	Accuracy: 96.6%, 95%; prediction: >93%	Used cubic kernel and 8 key parameters
Neural Networks [27,28,29,51,84,85,86,87,88,89,90,91,92,93,94,95,96,97,98,99,100,101,102,103,104,105,106,107,108,109]	Corneal topography images (quantity varies by study)	Sensitivity: 94–100%, specificity: 97.6–100%	High performance across multiple studies
FPA-K-means (Unsupervised) [110]	6961 OCT-based topography	Accuracy: 96.03%, precision: 96.29%, recall: 96.06%, F-score: 96.17%, purity: 96.03%	Unsupervised, pre-labeling bias-free
Automated Tree-Based Classifier [81]	Topography parameters from 372 eyes	Sensitivity: 100%, specificity: 99.5%	High performance differentiating KC from non-KC
CNN + Scheimpflug [123]	Pentacam four-map color-coded display of 3218 eyes	Accuracies: 0.98 to 0.99 for tested classes/sets	Excellent KC, SKC, vs. non-KC eye discrimination
Soft Voting Ensemble Model [119]	None (used clinical data to recommend topography)	Sensitivity: 90.5% (internal), 96.4% (external)	Used for triage and screening
BESTi [120]	Pentacam (topography) of 2893 eyes	Sensitivity and specificity: 84.97%	Outperformed PRFI and BAD-D indices for SKC detection
RETICS-based AI Model [60]	None (used morpho-geometric parameters)	Sensitivity: 85% (validation), 95% (training)	Categorized severity; considered gender, HOAs, coma-like aberrations
Ectasia Status Index (ESI) [35]	3156 eyes selected from OCT	Sensitivity: 97.7%, specificity: 94.1%	Based on posterior corneal surface and thickness; grades KC severity
Gradient Boosting Decision Tree (GBDT) algorithm compared with residents [121]	OCT-based topography of 2018 cases	Accuracy: 95.53% vs. 93.55% (residents), AUC: 0.99	Outperformed ophthalmology residents
Deep Learning (adjusted age algorithm) [61]	274 OCT color-coded maps	Accuracy: 85%	Differentiates progressive from non-progressive KC
Naïve Bayes [114]	None	Not quantified	Used for patient subgroup stratification
Hierarchical Clustering [114]	None	Not quantified	Unsupervised clustering method
K-Nearest Neighbor [118]	3886 eyes using Scheimpflug-based corneal tomography + biomechanical assessments	99.8% sensitivity, 99.8% specificity, and 99.8% accuracy (left eye); 99.9% sensitivity, 99.4% specificity, and 99.8% accuracy (right eye)	Used for classification and subgrouping
GAN [122]	Not specified	Not quantified	Used for data augmentation and feature representation
SVM (progression prediction) [107]	695 eyes using corneal topography	Accuracy: 97.72%	Second highest accuracy with faster training/testing time
Neural Networks (progression prediction) [107]	695 eyes using corneal topography	Accuracy: 98.29%	Highest accuracy in predicting KC progression

**Table 2:** Summary of Fuch’s endothelial dystrophy models.

Model Type	Imaging Modality	Performance Metrics	Notes
DL model [137]	18,720 AS-OCT	Sensitivity = 99%, Specificity = 98%	Differentiated normal, early-stage, and late-stage FECD
DL model [137]	18,720 AS-OCT	Early-FECD: AUC = 0.997, sensitivity = 91%, specificity = 97% Late-FECD: AUC = 0.974, sensitivity up to 100%, specificity = 92% FECD vs. non-FECD: AUC = 0.998, sensitivity 99%, specificity 98%	High accuracy in staging and diagnosis of FECD
DL model [138]	158,220 AS-OCT	FECD: AUC = 1.0, F1 = 100% Keratoconus: AUC = 0.99, F1 = 98% DED: AUC = 0.99, F1 = 90%	Multi-disease diagnosis, including FECD
Hierarchical DL model [139]	5325 Slit lamp photography	AUC = 0.939 (FECD + other dystrophies), AUC range = 0.90 = 0.95 (retrospective), >0.91 (prospective)	Included FECD as part of broader corneal dystrophy classification
Semantic segmentation DL model [140]	1772 Slit lamp photography	Accuracy = 79–99%, sensitivity = 53–99%, specificity = 85–99%	Detected 10 anterior segment pathologies including FECD
CNN-based DL model [142]	383 Specular microscopy	Success = 98% of images, error = 2.5–5.7% vs. 7.5–18.3% (manual); manual only worked in 31–72% of images	Outperformed traditional methods
DL model [145]	775 Salience map images	Internal validation: AUC = 0.92, sensitivity = 0.86, specificity = 0.86 External validation: AUC = 0.82, sensitivity = 0.74, specificity = 0.74	Detected abnormal images across FECD
DL model [145]	775 Salience map images	Internal validation: AUC = 0.96, sensitivity = 0.91, specificity = 0.91 External validation: AUC = 0.77, sensitivity = 0.69, specificity = 0.68	Distinguished FECD from other diagnoses
DL model [145]	775 Widefield salience map images	AUC = 0.88, Sensitivity = 0.79, Specificity = 0.78	Detected ECD > 1000 cells/mm^2^ to diagnose FECD
Support Vector Machine [146]	Not specified (247 eyes in 3 mm group and 149 in 5 mm group)	3 mm pupil: accuracy = 92.8%, sensitivity = 96.9%, precision = 94.8–95.2% 5 mm pupil: accuracy = 90.2–90.7%, sensitivity = 94.8%, precision = 92.1–92.7%	Differentiated healthy vs. pathological corneas using statistical features
DL model using edema fraction [147]	1992 AS-OCT	AUC = 0.97 overall; 0.96 (FECD vs. normal); 0.99 (non-FECD vs. normal)	EF used as early biomarker based on DCCT change
DL model using GAR% [147]	104 eyes from AS-OCT (central cornea)	Correlation = 0.60, *p* < 0.001	GAR% correlated with m-Krachmer grading scale

**Table 3:** Summary of dry eye disease models.

AI Model	Imaging Modality	Performance Metrics	Notes
NIPALS+ MLP Neural Network (MLP NN) [153]	None; used tear proteomics	Accuracy: 89.3%	Classified non-DED, DED, and MGD-associated DED using proteomics
Multi-layer Feed-forward NN [153]	None; used seven-biomarker tear panel	AUC: 0.93	Strong performance but limited interpretability
Hierarchical Clustering + RF [153]	None; used tear proteins and metabolites	Not specified	Differentiated DED from other OSDs; identified subgroups and risk factors
Infrared Thermography [154]	Thermal images (quantity not specified)	Sensitivity: 84–99.9%, specificity: 83–99.4%, accuracy: 84–99.8%	IRT + ML (KNN, PNN, SVM); high accuracy for ADDE classification
K-Nearest Neighbor [154]	Thermography images (quantity not specified)	Accuracy: 99.88%, sensitivity: 99.7%, specificity: 100%	Evaluated corneal temperature profiles
Artificial Neural Network [156]	None; tear proteomics (electrophoresis)	Accuracy: 89%	Automatically detected DED from protein electrophoretic patterns
Protein Chip Array + AI [157]	None; tear protein microarrays	Sensitivity and specificity: 90%	Differentiated DED patients from non-DED controls
EyeScan Algorithm [158]	8 slit lamp videos (TBUT)	Accuracy: 91%	First automated video-based DED tool (2007), expanded with additional tear metrics
CNN (FTBUT) [159]	60 fluorescein TBUT videos	Accuracy: 98.3%	Segmented TBUT break-up areas automatically
TBUT Video Algorithm [160]	30 TBUT video recordings	Accuracy: 83%	Diagnosed and graded DED severity based on TBUT
CNN (SPK detection) [161]	5160 fluorescein-stained images	Accuracy: 97%, r = 0.81 (with clinical grading)	Graded punctate dots and correlated with clinical severity
Lipid Interferometer + ML [162,163]	414 [163] and 138 [164], tear film lipid interferometer images	Agreement: 0.82	Classified healthy, ADDE, or evaporative DED
DL-based Blink Analysis [164,165]	Video recordings from 100 eyes [165] and 1196 frame images from a keratograph [166]	Not quantified	Linked blink frequency and incomplete blinking to DED
U-Net + ResNet [166]	Blink video recordings (quantity not specified)	Segmentation accuracy: 96.3%, classification accuracy: 96.0%	Differentiated partial/complete blinks; identified blink dynamics in DED
Random Forest Regression [167]	AS-OCT epithelial mapping of 114 patients	Sensitivity: 86.4%, specificity: 91.7%, AUC: 0.87	Identified epithelial thickness differences as DED markers
SANDE + NIBUT Rule-Based Algorithm [168]	Clinical survey + NIBUT (non-invasive TBUT) of 235 patients using infrared meibography	Sensitivity: 86%, specificity: 94%	Combined symptom and tear stability data for fast DED screening
General AI Models (Meta-analysis) [169]	Various	Accuracy: 91.91%, sensitivity: 89.58%, specificity: 92.62%	Based on pooled results across studies

**Table 4:** Summary of tear film models.

AI Model	Imaging Modalities	Performance Metrics	Notes
Automated Tear Film Thickness Model [170]	High-Resolution OCT (quantity not specified)	Relative accuracy: 65%	Fully automated; high reproducibility for DED diagnosis and treatment monitoring
CNN-Based TMH Segmentation [171]	485 Tear Meniscus Images (OCT)	IoU: 82.5%, correlation with ground truth: 0.965 vs. manual 0.898	Automated measurement of TMH; improved consistency and reliability in aqueous-deficient DED
Conventional Image Processing [172]	384 Ultrahigh-Resolution OCT images	Significant correlation (all r ≥ 0.657)	Accurately segmented TMH, tear meniscus area, depth, radius using traditional methods
Thresholding-Based ML Algorithm [173]	6658 AS-OCT	Sensitivity: 96%, specificity: 100%	Robust segmentation of lower tear meniscus; enhances objective quantification for DED evaluation

**Table 5:** Summary of infectious keratitis models.

Model Type	Imaging Modality	Performance Metrics	Notes
CNN (3 models) [183]	9329 slit lamp photos	Accuracies: 99.3% (IK), ~84% (BK vs. FK), 77.5% (yeast vs. filamentous)	Multi-task model for IK diagnosis and etiology differentiation
DeepIK (custom DL) [185]	23,055 slit lamp photos	Cohen’s Kappa: 0.70–0.77; time/image: 0.034 sec	Two-stage classification mimicking expert workflow; outperformed other DL models
Neural networks [188]	None (input clinical variables)	Accuracy: 90.7%	Early AI model distinguishing BK vs. FK; outperformed clinicians
Ensemble CNN [190,191]	2167 slit lamp photos	Sensitivity: 0.77; F1: 0.83; AUPRC: 0.904	BK vs. FK classification
CNN [190]	1330 cropped images	Accuracy: 80%	BK and FK classification
MobileNet [191]	980 slit lamp images	AUC: 0.86 (single-center), 0.83 (multi-center)	CNN comparison for BK/FK detection
ResNet-50 [192]	Slit lamp images (quantity not specified)	Specificity/sensitivity (BK): 80%/70%; (FK): 70%/80%	Differentiating BK vs. FK
NLP algorithm [193]	None (qualitative phrases from notes)	Sensitivity: 50–100%	Quantified MK features: centrality, thinning, depth
EfficientNet B3 [194]	1512 slit lamp images	Sensitivity: 74%; specificity: 64%	BK diagnosis; performance comparable to ophthalmologists
VGG19, ResNet50, DenseNet121 [189]	2167 mixed BK/FK images	Best: VGG19 F1 score: 0.78; AUPRC: 0.861	Comparative study for BK/FK classification
SLIT-Net, ResNeXt [195,196]	195 white light and 148 blue light slit lamp images [196]; 133 clinically suspected slit lamp images and 540 collated public-domain images [197]	Accuracy: up to 88.96%	Lesion segmentation models
R-CNN [197]	80 Slit lamp images	Dice coefficient: 0.74–0.76	Segmentation of IK features (e.g., infiltrates, hypopyons)
ResNet50 (multi-attribute) [198]	Slit lamp images (quantity not specified)	Accuracy: 89.51%	Multi-attribute IK feature detection
DenseNet + semi-automated algorithm [199,200,201]	288 Slit lamp photos [200]; 92 images [201]; not specified [202]	ICC: 0.96–0.98 (automated), 0.84–0.88 (human)	FK diagnosis; epithelial defect measurement
AlexNet, VGGNet + HMF [202]	977 abnormal and 876 normal slit lamp images	Accuracy: 99.95%	Used histogram matching fusion for enhancement
ARBP texture analysis [203]	183 normal and 195 abnormal confocal microscopy images	Accuracy: 99.74%; TPR, TNR, AUC ≈ 1	Texture-based classification
ResNet [204]	2088 in vivo confocal microscopy	AUC: 0.987; accuracy: 0.962; sensitivity: 0.918; specificity: 0.9834	FK classification
AlexNet, ZFNet, VGG16 [205]	Confocal microscopy (quantity not specified)	VGG16: accuracy: 0.992; sensitivity: 0.993; specificity: 0.992; AUC: 0.999	High performance in IK classification
DL models [140]	1772 and 5325 slit lamp images	AUCs > 0.91	Classification of IK subtypes and other corneal pathologies
Hybrid DL [206]	4306 images	Bacteria: 90.7%/0.963; fungi: 95.0%/0.975; Acanthamoeba: 97.9%/0.995; HSV: 92.3%/0.946	Multi-pathogen classification
CNN [207]	10,739 images	BK: 91.91%; FK: 79.77%; Acanthamoeba: 81.27%	Multi-class classification study
AlexNet, VGG-16, VGG-19 [208]	928 slit lamp images	Training: ~100%; testing: 99.1–100%	IK classification across three networks
Sequential CNN [209]	362 images	BK: 78.7%; FK: 74.23%; HSK: 75.1%	Outperformed 121 ophthalmologists
DenseNet121 + VCM [210]	3319 slit lamp images	F1: BK 0.431; FK 0.872; HSK 0.651	Pixel-level saliency-enhanced classification
InceptionV3 [211]	5673 slit lamp images	DenseNet121 outperformed ResNet50 and InceptionV3	Normal vs. BK, FK, HSK classification
DenseNet [212]	307 slit lamp images	Accuracy: 72%; AUC: 0.73; sensitivity: 69.6%; specificity: 76.5%	HSV necrotizing stromal keratitis classification
CNN [186]	3312 in vivo confocal microscopy (HRT3)	Accuracy, sensitivity, specificity: 76%	Acanthamoeba keratitis diagnosis
Logistic regression, RF, DT, Lasso [184]	1047 slit lamp images	Internal validation: mean AUC of 0.916, 0.920, and 0.859, respectively	Explored for FK diagnosis and clinical sign prediction
Systematic review [181,182]	34,070 slit lamp photos; 136,401 anterior segment photos, AS-OCT, IVCM, and corneal topography	Accuracy: 64.38% (BK vs. FK); 96.6% (infectious vs. noninfectious)	DL models outperformed clinicians; IVCM superior to ASP

**Table 6:** Summary of corneal neuropathy models.

AI Model	Image Modality	Performance Metrics	Diagnostic Target
Modified ResNet-50 [218]	369 confocal microscopy images	Sensitivity 1.0 for healthy controls, 0.85 for DPN+, 0.83 for DPN-	Diabetic peripheral neuropathy (DPN)
U-Net + adaptive neuro-fuzzy inference system [219]	Trained on 174 confocal microscopy images, validated with 534	AUC 0.95 (92% sensitivity, 80% specificity) for DPN detection	Diabetic neuropathy (DPN)
CNN model [220]	600 confocal microscopy images	Sensitivity 0.98, specificity 0.96, accuracy 0.97	DPN detection
Automatic segmentation model [221]	1698 confocal microscopy images	Sensitivity 0.68, specificity 0.87, AUC 0.83	DPN detection
Deep Grading CNN [222]	354 confocal microscopy images	85.64% accuracy	Nerve tortuosity quantification

**Table 7:** Summary of conjunctiva models.

Model Type	Imaging Modality	Performance Metrics	Notes
CNN [224]	115 segmented conjunctiva images	77.58% sensitivity (anemia detection)	Compared to lab test results for anemia detection
ANN [225]	271 color digital images of conjunctiva	0.89 correlation with ground truth	Evaluated conjunctival vascularity; best method out of two
Attention U-Net [226]	15 conjunctival images with motion artifacts	High segmentation performance	Developed for motion correction and segmentation in blurred images
Custom Algorithm [227]	101 vessels from 12 digital photographs	High intra-session repeatability; strong agreement with manual assessment	Measured conjunctival vessel width with minor estimation errors
Neural Network (JOAS criteria) [228]	5008 conjunctival images	71.8% vessel area detection; r = 0.737, *p* < 0.01	Graded conjunctival hyperemia severity vs. expert evaluation
Neural Network [229]	611 conjunctival images	75.1% accuracy; 78.7% sensitivity; 69.0% specificity	Detected diabetes via conjunctival microcirculation analysis
DL algorithms [231]	AS SS-OCT images (quantity not specified)	Near-perfect sensitivity and specificity	Applied to angle closure glaucoma detection and VA prediction; future potential in conjunctival tumor assessment
CNN with data augmentation [232]	398 smartphone-captured conjunctival images	97% accuracy	Detected conjunctival melanoma using enhanced training dataset
YOLOv5-based DL model [233]	6442 smartphone slit lamp images	AUC: 0.997 (ocular surface tumors)	High diagnostic accuracy for multiple ocular conditions
CorneAI platform [234,235]	5270 smartphone + slit lamp images	Accuracy: 86%; improved accuracy from 79.2% to 88.8% overall; AUC: 0.62 (tumor), 0.71 (deposits)	Improved attending and resident performance
ResNet50V2, YOLOv8x, VGG19 [236,237]	2774 IVCM images	Accuracy > 97%, precision ≥ 98%, recall ≥ 85%, F1 score ≥ 92%	Applied to OSSN; enabled subtype stratification
OSPM-enhanced classification model [238]	1455 tumor images	AUC range: 0.89–0.99 across datasets	Outperformed standard CNNs; matched senior ophthalmologists; improved junior diagnostic performance
MobileNetV2, NASNet, GoogleNet, ResNet50, InceptionV3 [239]	398 public ocular surface images	Best: MobileNetV2 AUC = 0.976, accuracy = 96.5%; with GANs: AUC = 0.983, accuracy = 97.2%	GAN-enhanced training improved results
Ensemble DL model [242]	172 slit lamp images	Accuracy = 94.12%, AUC = 0.980	Pterygium detection
VGG16 [243]	734 slit lamp images	Accuracy = 99%, sensitivity = 98%, specificity = 99.33%, Kappa = 0.98, F1 = 99%	High performance in binary classification
CNN-based segmentation model [244]	489 slit lamp images	Dice = 0.9620 (cornea), 0.9020 (pterygium); Kappa = 0.918	Effective in anatomical segmentation tasks
DL model [245]	258 slit lamp images	Sensitivity, specificity, and accuracy = 91.7%; F1 = 0.846	Classified primary, recurrent, and no pterygium
RFRC and SRU-Net [246]	20,987 slit lamp + 1094 smartphone images	Accuracy = 95.24%; fusion segmentation: F1 = 0.8981, sensitivity = 0.8709, specificity = 0.9668, AUC = 0.9295	Performed well across devices; comparable to clinicians
Two custom DL algorithms [247]	2503 slit lamp photographs	Any pterygium AUC = 99.5% (internal), 99.1% (ext1), 99.7% (ext2); referable AUC = 98.5%, 99.7%, 99.0%	High generalizability across datasets
MobileNetV2 [248]	436 slit lamp images	Sensitivity = 0.8370, specificity = 0.9048, F1 = 0.8250	Consistent high performance in multiple studies
MOSAIC (GPT-4 Turbo, Claude-3 Opus, Gemini-1.5 Pro) [249]	375 smartphone-acquired images	Accuracy = 86.96% (detection), 66.67% (grading pterygium)	Integrates LLMs in diagnostics
ML (with SHAP analysis) [250]	58 RNA sequencing samples	Performance dropped without APOE gene	Predicted corneal involvement in infectious conjunctivitis; identified APOE as key biomarker

**Table 8:** Summary of LLM diagnostic performance.

Model	Diagnostic Accuracy	Accuracy by Disease Type	AI–Physician Agreement			Physician–Physician Agreement
			Pairwise Agreement with Experts [253]	Interobserver Agreement [251,252]	Kappa (*p*-Value) [254]	
ChatGPT-4.0 (GPT-4.0)	85%, 85%, 80%	Degenerative: 100%, Inflammatory: 40%, Congenital: 83.3%, Infectious: 100%	80%, 75%, 85%, 80%	85%, 80%, 75%, 65% (vs. GPT-3.5)	0.348 (*p* = 0.040)	93.3%, 90.5%
ChatGPT-01	85%	—	—	8.3% less than physician average	—	93.3%
ChatGPT-3.5 (GPT-3.5)	60%, 60%	Degenerative: 66.7%, Inflammatory: 60%, Congenital: 50%, Infectious: 66.7%	60%, 60%, 60%, 60%	60%, 65% (vs. GPT-4.0)	0.146 (*p* = 0.209)	93.3%
GPT-4.0 Mini	55%	Degenerative: 83.3%, Inflammatory: 40%, Congenital: 33.3%, Infectious: 66.7%	55%, 50%, 60%, 65%	—	0.121 (*p* = 0.257)	90.5%
DeepSeek (V3 and R1)	90% (V3), 65% (R1)	Degenerative: 100%, Inflammatory: 40%, Congenital: 50%, Infectious: 66.7%	65%, 75%, 70%, 70%	3.3% less than physician average (V3)	0.178 (*p* = 0.162) (R1)	93.3%
Claude 3.5 Sonnet	70%	Degenerative: 100%, Inflammatory: 60%, Congenital: 50%, Infectious: 66.7%	60%, 60%, 60%, 65%	—	0.219 (*p* = 0.117)	90.5%
Grok3	70%	Degenerative: 83.3%, Inflammatory: 40%, Congenital: 66.7%, Infectious: 100%	80%, 75%, 85%, 80%	—	0.219 (*p* = 0.117)	90.5%
Qwen 2.5 MAX	55%	—	—	38.3% less than physician average	—	93.3%
Gemini 1.5 Flash	30%	Degenerative: 33.3%, Inflammatory: 0%, Congenital: 33.3%, Infectious: 66.7%	30%, 30%, 30%, 30%	—	0.044 (*p* = 0.502)	90.5%

## Data Availability

No new data were created or analyzed in this study.

## References

[1] Steinmetz J.D., GBD 2019 Blindness and Vision Impairment Collaborators, Vision Loss Expert Group of the Global Burden of Disease Study (2021). Causes of blindness and vision impairment in 2020 and trends over 30 years, and prevalence of avoidable blindness in relation to VISION 2020: The Right to Sight: An analysis for the Global Burden of Disease Study. Lancet Glob. Health.

[2] Burton M.J., Ramke J., Marques A.P., Bourne R.R.A., Congdon A., Jones I., Tong B., Arunga S., Bachani D., Bascaran C. (2021). The Lancet Global Health Commission on Global Eye Health: Vision beyond 2020. Lancet Glob. Health.

[3] Whitcher J.P., Srinivasan M., Upadhyay M.P. (2001). Corneal blindness: A global perspective. Bull. World Health Organ..

[4] Morthen M.K., Magno M.S., Utheim T.P., Snieder H., Hammond C.J., Vehof J. (2021). The physical and mental burden of dry eye disease: A large population-based study investigating the relationship with health-related quality of life and its determinants. Ocul. Surf..

[5] Lanza M., Incagli F., Ceccato C., Reffo M.E., Mercuriali E., Parmeggiani F., Pagliano E., Saletti V., Leonardi M., Suppiej A. (2024). Quality of life, functioning and participation of children and adolescents with visual impairment: A scoping review. Res. Dev. Disabil..

[6] Kandel H., Pesudovs K., Watson S.L. (2020). Measurement of Quality of Life in Keratoconus. Cornea.

[7] Yu J., Asche C.V., Fairchild C.J. (2011). The economic burden of dry eye disease in the United States: A decision tree analysis. Cornea.

[8] Collier S.A., Gronostaj M.P., MacGurn A.K., Cope J.R., Awsumb K.L., Yoder J.S., Beach M.J. (2014). Estimated burden of keratitis—United States, 2010. MMWR Morb. Mortal. Wkly. Rep..

[9] Vashist P., Gupta N., Tandon R., Gupta S.K., Dwivedi S., Mani K. (2016). Population-based assessment of vision-related quality of life in corneal disease: Results from the CORE study. Br. J. Ophthalmol..

[10] Baral P., Kumaran S., Stapleton F., Pesudovs K. (2024). Quality of life impacts of ocular surface diseases: A qualitative exploration. Investig. Ophthalmol. Vis. Sci..

[11] Cabrera-Aguas M., Khoo P., Watson S.L. (2022). Infectious keratitis: A review. Clin. Exp. Ophthalmol..

[12] Han S.B., Liu Y.-C., Mohamed-Noriega K., Mehta J.S. (2021). Advances in Imaging Technology of Anterior Segment of the Eye. J. Ophthalmol..

[13] Ang M., Baskaran M., Werkmeister R.M., Chua J., Schmidl D., Aranha dos Santos V., Garhofer G., Mehta J.S., Schmetterer L. (2018). Anterior segment optical coherence tomography. Prog. Retin. Eye Res..

[14] Kojima T., Dogru M., Kawashima M., Nakamura S., Tsubota K. (2020). Advances in the diagnosis and treatment of dry eye. Prog. Retin. Eye Res..

[15] Cheng K.K.W., Fingerhut L., Duncan S., Prajna N.V., Rossi A.G., Mills B. (2024). In vitro and ex vivo models of microbial keratitis: Present and future. Prog. Retin. Eye Res..

[16] Tuft S., Somerville T.B., Li J.-P.O., Neal T., De S., Horsburgh M.J., Fothergill J.L., Foulkes D., Kaye S. (2022). Bacterial keratitis: Identifying the areas of clinical uncertainty. Prog. Retin. Eye Res..

[17] Ting D.S.W., Peng L., Varadarajan A.V., Keane P.A., Burlina P.M., Chiang M.F., Schmetterer L., Pasquale L.R., Bressler N.M., Webster D.R. (2019). Deep learning in ophthalmology: The technical and clinical considerations. Prog. Retin. Eye Res..

[18] Haug C.J., Drazen J.M. (2023). Artificial Intelligence and Machine Learning in Clinical Medicine, 2023. N. Engl. J. Med..

[19] Rampat R., Deshmukh R., Chen X., Ting D.S.W., Said D.G., Dua H.S., Ting D.S.J. (2021). Artificial Intelligence in Cornea, Refractive Surgery, and Cataract: Basic Principles, Clinical Applications, and Future Directions. Asia Pac. J. Ophthalmol..

[20] Nguyen T., Ong J., Masalkhi M., Waisberg E., Zaman N., Sarker P., Aman S., Lin H., Luo M., Ambrosio R. (2024). Artificial intelligence in corneal diseases: A narrative review. Cont. Lens Anterior Eye.

[21] Kang L.D., Ballouz D., Woodward M.A. (2022). Artificial intelligence and corneal diseases. Curr. Opin. Ophthalmol..

[22] Ting D.S.J., Foo V.H.X., Yang L.W.H., Sia J.T., Ang M., Lin H., Chodosh J., Mehta J.S., Ting D.S.W. (2021). Artificial intelligence for anterior segment diseases: Emerging applications in ophthalmology. Br. J. Ophthalmol..

[23] Linde G., Rodrigues de Souza W., Chalakkal R., Danesh-Meyer H.V., O’Keeffe B., Hong S.C. (2024). A comparative evaluation of deep learning approaches for ophthalmology. Sci. Rep..

[24] Li Z., Jiang J., Chen K., Chen Q., Zheng Q., Liu X., Weng H., Wu S., Chen W. (2021). Preventing corneal blindness caused by keratitis using artificial intelligence. Nat. Commun..

[25] Biousse V., Newman N.J., Najjar R.P., Vasseneix C., Xu X., Ting D.S., Milea L.B., Hwang J.-M. (2020). Kim, D.H.; Yang, H.K.; et al. Optic Disc Classification by Deep Learning versus Expert Neuro-Ophthalmologists. Ann. Neurol..

[26] Yoo T.K., Ryu I.H., Lee G., Kim Y., Kim J.K., Lee I.S., Kim J.S., Rim T.H. (2019). Adopting machine learning to automatically identify candidate patients for corneal refractive surgery. NPJ Digit. Med..

[27] Accardo P.A., Pensiero S. (2002). Neural network-based system for early keratoconus detection from corneal topography. J. Biomed. Inform..

[28] Kamiya K., Ayatsuka Y., Kato Y., Fujimura F., Takahashi M., Shoji N., Mori Y., Miyata K. (2019). Keratoconus detection using deep learning of colour-coded maps with anterior segment optical coherence tomography: A diagnostic accuracy study. BMJ Open.

[29] Souza M.B., de Medeiros F.W., Souza D.B., Alves M.R. (2008). Detection of keratoconus based on a neural network with orbscan. Arq. Bras. Oftalmol..

[30] Maeda N., Klyce S.D., Smolek M.K., Thompson H.W. (1994). Automated keratoconus screening with corneal topography analysis. Investig. Ophthalmol. Vis. Sci..

[31] Malyugin B., Sakhnov S., Izmailova S., Boiko E., Pozdeyeva N., Axenova L., Axenov K., Titov A., Terentyeva A., Zakaraiia T. (2021). Keratoconus Diagnostic and Treatment Algorithms Based on Machine-Learning Methods. Diagnostics.

[32] Arbelaez M.C., Versaci F., Vestri G., Barboni P., Savini G. (2012). Use of a support vector machine for keratoconus and subclinical keratoconus detection by topographic and tomographic data. Ophthalmology.

[33] Toutounchian F., Shanbehzadeh J., Khanlari M. Detection of Keratoconus and Suspect Keratoconus by Machine Vision. Proceedings of the International Multiconference of Engineers and Computer Scientists, IMECS 2012.

[34] Song P., Ren S.W., Liu Y., Li P., Zeng Q.Y. (2022). Detection of subclinical keratoconus using a novel combined tomographic and biomechanical model based on an automated decision tree. Sci. Rep..

[35] Yousefi S., Yousefi E., Takahashi H., Hayashi T., Tampo H., Inoda S., Arai Y., Asbell P. (2018). Keratoconus severity identification using unsupervised machine learning. PLoS ONE.

[36] Klyce S.D., Smolek M.K., Maeda N. (2000). Keratoconus detection with the KISA% method—Another view. J. Cataract Refract. Surg..

[37] Jimenez-Garcia M., Issarti I., Kreps E.O., Dhubhghaill S.N., Koppen C., Varssano D., Rozema J.J. (2021). Keratoconus progression forecast by means of a time delay neural network. Investig. Ophthalmol. Vis. Sci..

[38] Falahati Marvast F., Arabalibeik H., Alipour F., Sheikhtaheri A., Nouri L., Soozande M., Yarmahmoodi M. (2016). Evaluation of RGP Contact Lens Fitting in Keratoconus Patients Using Hierarchical Fuzzy Model and Genetic Algorithms. Medicine Meets Virtual Reality 22.

[39] Hu D., Lin Z., Jiang J., Li P., Zhang Z., Yang C. (2022). Identification of Key Genes and Molecular Pathways in Keratoconus: Integrating Text Mining and Bioinformatics Analysis. BioMed Res. Int..

[40] Marsack J.D., Benoit J.S., Kollbaum P.S., Anderson H.A. (2019). Application of Topographical Keratoconus Detection Metrics to Eyes of Individuals with Down Syndrome. Optom. Vis. Sci..

[41] Wang L., Wang Y., Liu J., Zhao W. (2022). Identification of Important Genes of Keratoconus and Construction of the Diagnostic Model. Genet. Res..

[42] Kamiya K., Ayatsuka Y., Kato Y., Shoji N., Miyai T., Ishii H., Mori Y., Miyata K. (2021). Prediction of keratoconus progression using deep learning of anterior segment optical coherence tomography maps. Ann. Transl. Med..

[43] Castro-Luna G., Jimenez-Rodriguez D., Castano-Fernandez A.B., Perez-Rueda A. (2021). Diagnosis of Subclinical Keratoconus Based on Machine Learning Techniques. J. Clin. Med..

[44] Wisse R.P.L., Godefrooij D.A., Nuijts R. (2019). Using Machine Learning to Monitor Keratoconus Progression—Reply. JAMA Ophthalmol..

[45] Yucekul B., Dick H.B., Taneri S. (2022). Systematic Detection of Keratoconus in Optical Coherence Tomography: Corneal and Epithelial Thickness Maps. J. Cataract Refract. Surg..

[46] Reinstein D.Z., Archer T.J., Urs R., Gobbe M., RoyChoudhury A., Silverman R.H. (2015). Detection of Keratoconus in Clinically and Algorithmically Topographically Normal Fellow Eyes Using Epithelial Thickness Analysis. J. Refract. Surg..

[47] Ahuja A.S., Halperin L.S. (2019). Using Machine Learning to Monitor Keratoconus Progression. JAMA Ophthalmol..

[48] Kato N., Masumoto H., Tanabe M., Sakai C., Negishi K., Torii H., Tabuchi H., Tsubota K. (2021). Predicting Keratoconus Progression and Need for Corneal Crosslinking Using Deep Learning. J. Clin. Med..

[49] Kalin N.S., Maeda N., Klyce S.D., Hargrave S., Wilson S.E. (1996). Automated Topographic Screening for Keratoconus in Refractive Surgery Candidates. CLAO J..

[50] Kheiri S., Meshkani M.R., Faghihzadeh S. (2005). A Correlated Frailty Model for Analysing Risk Factors in Bilateral Corneal Graft Rejection for Keratoconus: A Bayesian Approach. Stat. Med..

[51] Smolek M.K., Klyce S.D. (1996). Neural Network Pair for the Detection and Grading of Keratoconus and Keratoconus Suspects by Videokeratoscopy. Investig. Ophthalmol. Vis. Sci..

[52] Dong Y., Li D., Guo Z., Liu Y., Lin P., Lv B., Lv C., Xie G., Xie L. (2021). Dissecting the Profile of Corneal Thickness with Keratoconus Progression Based on Anterior Segment Optical Coherence Tomography. Front. Neurosci..

[53] Fariselli C., Vega-Estrada A., Arnalich-Montiel F., Alio J.L. (2020). Artificial Neural Network to Guide Intracorneal Ring Segments Implantation for Keratoconus Treatment: A Pilot Study. Eye Vis..

[54] Hallett N., Yi K., Dick J., Hodge C., Sutton G., Wang Y.G., You J. Deep Learning Based Unsupervised and Semi-supervised Classification for Keratoconus. Proceedings of the 2020 International Joint Conference on Neural Networks (IJCNN).

[55] Hasan S.A., Singh M. An Algorithm to Differentiate Astigmatism from Keratoconus in Axial Topgraphic Images. Proceedings of the 2015 International Conference on Industrial Instrumentation and Control (ICIC).

[56] Silverman R.H., Urs R., RoyChaudhury A., Archer T.J., Gobbe M., Reinstein D.Z. (2014). Epithelial Remodeling as Basis for Machine-Based Identification of Keratoconus. Investig. Ophthalmol. Vis. Sci..

[57] Souza M.B., Medeiros F.W., Souza D.B., Garcia R., Alves M.R. (2010). Evaluation of Machine Learning Classifiers in Keratoconus Detection from Orbscan II Examinations. Clinics.

[58] Subramanian P., Ramesh G.P., Parameshachari B.D. (2022). Comparative Analysis of Machine Learning Approaches for the Early Diagnosis of Keratoconus. Distributed Computing and Optimization Techniques, ICDCOT 2021.

[59] Valdes-Mas M.A., Martin-Guerrero J.D., Ruperez M.J., Pastor F., Dualde C., Monserrat C., Peris-Martinez C. (2014). A New Approach Based on Machine Learning for Predicting Corneal Curvature (K1) and Astigmatism in Patients with Keratoconus after Intracorneal Ring Implantation. Comput. Methods Programs Biomed..

[60] Bolarin J.M., Cavas E., Velazquez J.S., Alio J.L. (2020). A Machine-Learning Model Based on Morphogeometric Parameters for RETICS Disease Classification and GUI Development. Appl. Sci..

[61] Kato N., Masumoto H., Tanabe M., Sakai C., Negishi K., Torii H., Tabuchi H., Tsubota K. (2021). Predicting keratoconus progression using deep learning. Investig. Ophthalmol. Vis. Sci..

[62] Cao K., Verspoor K., Chan E., Daniell M., Sahebjada S., Baird P.N. (2021). Machine learning with a reduced dimensionality representation of comprehensive Pentacam tomography parameters to identify subclinical keratoconus. Comput. Biol. Med..

[63] Cao K., Verspoor K., Chan E., Daniell M., Sahebjada S., Baird P.N. (2021). Novel, high-performance machine learning model for detection of subclinical keratoconus. Investig. Ophthalmol. Vis. Sci..

[64] Cao K., Verspoor K., Sahebjada S., Baird P.N. (2020). Evaluating the Performance of Various Machine Learning Algorithms to Detect Subclinical Keratoconus. Transl. Vis. Sci. Technol..

[65] Gao Y.H., Wu Q., Li J., Sun J.D., Wan W.B. SVM-Based Automatic Diagnosis Method for Keratoconus. Proceedings of the Second International Workshop on Pattern Recognition.

[66] Lavric A., Anchidin L., Popa V., Al-Timemy A.H., Alyasseri Z., Takahashi H., Yousefi S., Hazarbassanov R.M. (2021). Evaluation of keratoconus detection from elevation, topography and pachymetry raw data using machine learning. Investig. Ophthalmol. Vis. Sci..

[67] Herber R., Spoerl E., Pillunat L.E., Raiskup F. (2020). Classification of dynamic corneal response parameters concerning the topographical severity of keratoconus using the dynamic Scheimpflug imaging and machine-learning algorithms. Investig. Ophthalmol. Vis. Sci..

[68] Hernandez L.A., Sanchez-Huerta V., Ramirez-Fernandez M., Hernandez-Quintela E. (2020). Combinatorial approach to determine top performing keratometric features and machine learning algorithms for keratoconus detection. Investig. Ophthalmol. Vis. Sci..

[69] Hidalgo I.R., Perez P.R., Rozema J.J., Tassignon M. (2014). Comparison of Machine Learning Methods to Automatically Classify Keratoconus. Investig. Ophthalmol. Vis. Sci..

[70] Issarti I., Consejo A., Jimenez-Garcia M., Hershko S., Koppen C., Rozema J.J. (2019). Computer aided diagnosis for suspect keratoconus detection. Comput. Biol. Med..

[71] Issarti I., Consejo A., Jimenez-Garcia M., Kreps E.O., Koppen C., Rozema J.J. (2020). Logistic index for keratoconus detection and severity scoring (Logik). Comput. Biol. Med..

[72] Kovacs I., Mihaltz K., Kranitz K., Juhasz E., Takacs A., Dienes L., Gergely R., Nagy Z. (2016). Accuracy of machine learning classifiers using bilateral data from a Scheimpflug camera for identifying eyes with preclinical signs of keratoconus. J. Cataract Refract. Surg..

[73] Langenbucher A., Hafner L., Eppig T., Seitz B., Szentmary N., Flockerzi E. (2021). Keratoconus detection and classification from parameters of the Corvis (R) ST A study based on algorithms of machine learning. Ophthalmologe.

[74] Lavric A., Anchidin L., Popa V., Al-Timemy A.H., Alyasseri Z., Takahashi H. (2021). Keratoconus Severity Detection From Elevation, Topography and Pachymetry Raw Data Using a Machine Learning Approach. IEEE Access.

[75] Lyra D., Ribeiro G., Torquetti L., Ferrara P., Machado A., Lyra J.M. (2018). Computational Models for Optimization of the Intrastromal Corneal Ring Choice in Patients With Keratoconus Using Corneal Tomography Data. J. Refract. Surg..

[76] Mehravaran S., Dehzangi I., Rahman M.M. (2021). Interocular Symmetry Analysis of Corneal Elevation Using the Fellow Eye as the Reference Surface and Machine Learning. Healthcare.

[77] Ruiz Hidalgo I., Rodriguez P., Rozema J.J., Ni Dhubhghaill S., Zakaria N., Tassignon M.-J., Koppen C. (2016). Evaluation of a Machine-Learning Classifier for Keratoconus Detection Based on Scheimpflug Tomography. Cornea.

[78] Ruiz Hidalgo I., Rozema J.J., Saad A., Gatinel D., Rodriguez P., Zakaria N., Koppen C. (2017). Validation of an Objective Keratoconus Detection System Implemented in a Scheimpflug Tomographer and Comparison with Other Methods. Cornea.

[79] Shi C., Wang M., Zhu T., Zhang Y., Ye Y., Jiang J., Chen S., Lu F., Shen M. (2020). Machine learning helps improve diagnostic ability of subclinical keratoconus using Scheimpflug and OCT imaging modalities. Eye Vis..

[80] Silverman R.H., Urs R., RoyChaudhury A., Archer T.J., Gobbe M., Reinstein D.Z. (2017). Combined tomography and epithelial thickness mapping for diagnosis of keratoconus. Eur. J. Ophthalmol..

[81] Smadja D., Touboul D., Cohen A., Doveh E., Santhiago M.R., Mello G.R., Krueger R.R., Colin J. (2013). Detection of subclinical keratoconus using an automated decision tree classification. Am. J. Ophthalmol..

[82] Srivatsa P.R., Shetty R., Matalia H., Roy A.S. (2015). A new Zernike algorithm to link asymmetric corneal thickness to corneal wavefront aberrations for diagnosis of keratoconus. Investig. Ophthalmol. Vis. Sci..

[83] Takahashi H., Al-Timemy A.H., Mosa Z.M., Alyasseri Z., Lavric A., Filho J.A.P.M., Yuda K., Hazarbassonov R.M., Yousefi S. (2021). Detecting keratoconus severity from corneal data of different populations with machine learning. Investig. Ophthalmol. Vis. Sci..

[84] Afifah A., Syafira F., Afladhanti P.M., Dharmawidiarini D. (2024). Artificial intelligence as diagnostic modality for keratoconus: A systematic review and meta-analysis. J. Taibah Univ. Med. Sci..

[85] Alshammari G., Hamad A.A., Abdullah Z.M., Alshareef A.M., Alhebaishi N., Alshammari A., Belay A. (2021). Applications of Deep Learning on Topographic Images to Improve the Diagnosis for Dynamic Systems and Unconstrained Optimization. Wirel. Commun. Mob. Comput..

[86] Al-Timemy A., Al-Zubaidi L., Ghaeb N., Takahashi H., Lavric A., Mosa Z., Hazarbassanov R.M., Alyasseri Z.A.A., Yousefi S. (2022). A device-agnostic deep learning model for detecting keratoconus based on anterior elevation corneal maps. Investig. Ophthalmol. Vis. Sci..

[87] Al-Timemy A.H., Ghaeb N.H., Mosa Z.M., Escudero J. (2022). Deep Transfer Learning for Improved Detection of Keratoconus using Corneal Topographic Maps. Cogn. Comput..

[88] Al-Timemy A.H., Hazarbassanov R.M., Mosa Z.M., Alyasseri M., Lavric A., Oliveira da Rosa C.A., Griz C.P., Takahashi H., Yousefi S. (2021). A hybrid deep learning framework for keratoconus detection based on anterior and posterior corneal maps. Investig. Ophthalmol. Vis. Sci..

[89] Al-Timemy A.H., Mosa Z.M., Alyasseri Z., Lavric A., Lui M.M., Hazarbassanov R.M., Yousefi S. (2021). A Hybrid Deep Learning Construct for Detecting Keratoconus from Corneal Maps. Transl. Vis. Sci. Technol..

[90] Dos Santos V.A., Schmetterer L., Stegmann H., Pfister M., Messner A., Schmidinger G., Garhofer G., Werkmeister R.M. (2019). CorneaNet: Fast segmentation of cornea OCT scans of healthy and keratoconic eyes using deep learning. Biomed. Opt. Express.

[91] Feng R.W., Xu Z., Zheng X., Hu H., Jin X., Chen D.Z., Yao K., Wu J. (2021). KerNet: A Novel Deep Learning Approach for Keratoconus and Sub-Clinical Keratoconus Detection Based on Raw Data of the Pentacam HR System. IEEE J. Biomed. Health Inform..

[92] Firat M., Cankaya C., Cinar A., Tuncer T. (2022). Automatic detection of keratoconus on Pentacam images using feature selection based on deep learning. Int. J. Imaging Syst. Technol..

[93] Fisher A.C., Taktak A.F.G., Iskander D.R., Naroo S.A., Shah S., Kaye S.B., Batterbury M. (2004). Optimisation by an Artificial Neural Network of general ellipsotoric, Fourier Series and Zernike polynomial decompositions of anterior and posterior Orbscan topography data in keratoconus. Investig. Ophthalmol. Vis. Sci..

[94] Gairola S., Joshi P., Balasubramaniam A., Murali K., Kwatra N., Jain M. Keratoconus Classifier for Smartphone-based Corneal Topographer. Proceedings of the Annual International Conference of the IEEE Engineering in Medicine & Biology Society.

[95] Gao H.B., Pan Z.-G., Shen M.-X., Lu F., Li H., Zhang X.-Q. (2022). KeratoScreen: Early Keratoconus Classification With Zernike Polynomial Using Deep Learning. Cornea.

[96] Ghaderi M., Sharifi A., Jafarzadeh Pour E. (2021). Proposing an ensemble learning model based on neural network and fuzzy system for keratoconus diagnosis based on Pentacam measurements. Int. Ophthalmol..

[97] Kamiya K., Ayatsuka Y., Kato Y., Shoji N., Mori Y., Miyata K. (2021). Diagnosability of Keratoconus Using Deep Learning with Placido Disk-Based Corneal Topography. Front. Med..

[98] Karimi A., Meimani N., Razaghi R., Rahmati S.M., Jadidi K., Rostami M. (2018). Biomechanics of the Healthy and Keratoconic Corneas: A Combination of the Clinical Data, Finite Element Analysis, and Artificial Neural Network. Curr. Pharm. Des..

[99] Kuo B.-I., Chang W.-Y., Liao T.-S., Liu F.-Y., Liu H.-Y., Chu H.-S., Chen W.-L., Hu F.-R., Yen J.-Y., Wang I.-J. (2020). Keratoconus Screening Based on Deep Learning Approach of Corneal Topography. Transl. Vis. Sci. Technol..

[100] Lavric A., Valentin P. (2019). KeratoDetect: Keratoconus Detection Algorithm Using Convolutional Neural Networks. Comput. Intell. Neurosci..

[101] Dongfang L., Yanling D., Sen X., Zhen G., Suxia L., Yan G., Bin L., Lixin X. (2021). Deep learning based lesion detection from anterior segment optical coherence tomography images and its application in the diagnosis of keratoconus. Chin. J. Ophthalmol..

[102] Liu H., Anwar M., Koaik M., Taylor S., Karanjia R., Mintsioulis G., Ziai S., Baig K. (2021). Deep Learning for Detection of Keratoconus and Prediction of Crosslinking Efficacy. Investig. Ophthalmol. Vis. Sci..

[103] Pavlatos E., Huang D., Li Y. (2022). Combining OCT Corneal Topography and Thickness Maps to Diagnose Keratoconus Using a Convolutional Neural Network. Investig. Ophthalmol. Vis. Sci..

[104] Perissutti P., Accardo A.P., Pensiero S., Salvetat M.L. Automatic keratoconus detection by means of a neural network: Comparison between a monocular and a binocular approach. Proceedings of the 20th Annual International Conference of the IEEE Engineering in Medicine and Biology Society Biomedical Engineering Towards the Year 2000 and Beyond.

[105] Quah X.M., Kerdraon Y. (2022). Construction and evaluation of an artificial neural network based screening tool for keratoconus using refractive error measurements. Clin. Exp. Ophthalmol..

[106] Smolek M.K., Klyce S.D. (1997). Current keratoconus detection methods compared with a neural network approach. Investig. Ophthalmol. Vis. Sci..

[107] Ucar M., Sen B., Cakmak H.B., IEEE A Novel Classification and Estimation Approach for Detecting Keratoconus Disease with Intelligent Systems. Proceedings of the 2013 8th International Conference on Electrical and Electronics Engineering (ELECO).

[108] Zaki W., Daud M.M., Saad A.H., Hussain A., Mutalib H.A. (2021). A Mobile Solution for Lateral Segment Photographed Images Based Deep Keratoconus Screening Method. Int. J. Integr. Eng..

[109] Zaki W., Daud M.M., Saad A.H., Hussain A., Mutalib H.A. Towards Automated Keratoconus Screening Approach using Lateral Segment Photographed Images. Proceedings of the 2020 IEEE-EMBS Conference on Biomedical Engineering and Science (IECBES 2020): Leading Modern Healthcare Technology Enhancing Wellness.

[110] Hazarbassanov R.M., Alyasseri Z.A.A., Al-Timemy A., Lavric A., Abasid A.K., Takahashi H., Filho J.A.M., Campos M., Yousefi S. (2022). Detecting keratoconus on two different populations using an unsupervised hybrid artificial intelligence model. Investig. Ophthalmol. Vis. Sci..

[111] Lavric A., Popa V., Takahashi H., Yousefi S. (2020). Detecting Keratoconus From Corneal Imaging Data Using Machine Learning. IEEE Access.

[112] Herber R., Pillunat L.E., Raiskup F. (2021). Development of a classification system based on corneal biomechanical properties using artificial intelligence predicting keratoconus severity. Eye Vis..

[113] Aatila M., Lachgar M., Hamid H., Kartit A. (2021). Keratoconus Severity Classification Using Features Selection and Machine Learning Algorithms. Comput. Math. Methods Med..

[114] Hosoda Y., Miyake M., Meguro A., Tabara Y., Iwai S., Ueda-Arakawa N., Nakano E., Mori Y., Yoshikawa M., Nakanishi H. (2020). Keratoconus-susceptibility gene identification by corneal thickness genome-wide association study and artificial intelligence IBM Watson. Commun. Biol..

[115] Vandevenne M.M., Favuzza E., Veta M., Lucenteforte E., Berendschot T.T., Mencucci R., Nuijts R.M., Virgili G., Dickman M.M. (2023). Artificial intelligence for detecting keratoconus. Cochrane Database Syst. Rev..

[116] Niazi S., Jiménez-García M., Findl O., Gatzioufas Z., Doroodgar F., Shahriari M.H., Javadi M.A. (2023). Keratoconus Diagnosis: From Fundamentals to Artificial Intelligence: A Systematic Narrative Review. Diagnostics.

[117] Hashemi H., Doroodgar F., Niazi S., Khabazkhoob M., Heidari Z. (2024). Comparison of different corneal imaging modalities using artificial intelligence for diagnosis of keratoconus: A systematic review and meta-analysis. Graefes Arch. Clin. Exp. Ophthalmol..

[118] Rocha K.M., Van den Berg R., Van den Berg A., Ambrosio R. (2022). Optimized artificial intelligence for enhanced ectasia detection using Scheimpflug-based corneal tomography and biomechanical data. Am. J. Ophthalmol..

[119] Ahn H., Kim N.E., Chung J.L., Kim Y.J., Jun I., Kim T.-I., Seo K.Y. (2022). Patient Selection for Corneal Topographic Evaluation of Keratoconus: A Screening Approach Using Artificial Intelligence. Front. Med..

[120] Almeida G.C., Guido R.C., Balarin Silva H.M., Brandão C.C., de Mattos L.C., Lopes B.T., Machado A.P., Ambrósio R. (2022). New Artificial Intelligence Index Based on Scheimpflug Corneal Tomography to Distinguish Subclinical Keratoconus from Healthy Corneas. J. Cataract Refract. Surg..

[121] Zou H.H., Xu J.H., Zhang L., Ji S.F., Wang Y. (2019). Assistant Diagnose for Subclinical Keratoconus by Artificial Intelligence. Chin. J. Ophthalmol..

[122] Goodman D., Zhu A.Y. (2024). Utility of Artificial Intelligence in the Diagnosis and Management of Keratoconus: A Systematic Review. Front. Ophthalmol..

[123] Abdelmotaal H., Mostafa M.M., Mostafa A.N.R., Mohamed A.A., Abdelazeem K. (2020). Classification of Color-Coded Scheimpflug Camera Corneal Tomography Images Using Deep Learning. Trans. Vis. Sci. Technol..

[124] Gatinel D., Saad A. (2012). The Challenges of the Detection of Subclinical Keratoconus at Its Earliest Stage. Int. J. Keratoconus Ectatic Corneal Dis..

[125] Kanellopoulos A.J. (2021). Keratoconus Management With Customized Photorefractive Keratectomy by Artificial Intelligence Ray-Tracing Optimization Combined with Higher Fluence Corneal Crosslinking: The Ray-Tracing Athens Protocol. Cornea.

[126] Kundu G., Shetty R., Khamar P., Mullick R., Gupta S., Nuijts R., Roy A.S. (2021). Universal Architecture of Corneal Segmental Tomography Biomarkers for Artificial Intelligence-Driven Diagnosis of Early Keratoconus. Br. J. Ophthalmol..

[127] Lopes B.T., Ramos I.C., Salomao M.Q., Guerra F.P., Schallhorn S.C., Schallhorn J.M., Vinciguerra R., Vinciguerra P., Price F.W., Price M.O. (2018). Enhanced Tomographic Assessment to Detect Corneal Ectasia Based on Artificial Intelligence. Am. J. Ophthalmol..

[128] Mohammadpour M., Heidari Z., Hashemi H., Yaseri M., Fotouhi A. (2022). Comparison of Artificial Intelligence-Based Machine Learning Classifiers for Early Detection of Keratoconus. Eur. J. Ophthalmol..

[129] Shetty R., Kundu G., Narasimhan R., Khamar P., Gupta K., Singh N., Nuijits R.M.M.A., Roy A.S. (2021). Artificial Intelligence Efficiently Identifies Regional Differences in the Progression of Tomographic Parameters of Keratoconic Corneas. J. Refract. Surg..

[130] Tan Z., Chen X., Li K., Liu Y., Cao H., Li J., Jhanji V., Zou H., Liu F., Wang R. (2022). Artificial Intelligence-Based Diagnostic Model for Detecting Keratoconus Using Videos of Corneal Force Deformation. Transl. Vis. Sci. Technol..

[131] Xu Z., Feng R., Jin X., Hu H., Ni S., Xu W., Zheng X., Wu J., Yao K. (2022). Evaluation of Artificial Intelligence Models for the Detection of Asymmetric Keratoconus Eyes Using Scheimpflug Tomography. Clin. Exp. Ophthalmol..

[132] Niazi S., Doroodgar F., Hashemi Nazari S., Rahimi Y., Alió Del Barrio J.L., Gatzioufas Z., Findl O., Vinciguerra P., Vinciguerra R., Moshirfar M. (2024). Refractive Surgical Approaches to Keratoconus: A Systematic Review and Network Meta-Analysis. Surv. Ophthalmol..

[133] Niazi S., Gatzioufas Z., Doroodgar F., Findl O., Baradaran-Rafii A., Liechty J., Moshirfar M. (2024). Keratoconus: Exploring Fundamentals and Future Perspectives—A Comprehensive Systematic Review. Ther. Adv. Ophthalmol..

[134] Lu N.J., Koppen C., Hafezi F., Ní Dhubhghaill S., Aslanides I.M., Wang Q.M., Cui L.L., Rozema J.J. (2023). Combinations of Scheimpflug Tomography, Ocular Coherence Tomography and Air-Puff Tonometry Improve the Detection of Keratoconus. Contact Lens Anterior Eye.

[135] Bodmer N.S., Christensen D.G., Bachmann L.M., Faes L., Sanak F., Iselin K., Kaufmann C., Thiel M.A., Baenninger P.B. (2024). Deep Learning Models Used in the Diagnostic Workup of Keratoconus: A Systematic Review and Exploratory Meta-Analysis. Cornea.

[136] Gurnani B., Somani A.N., Moshirfar M., Patel B.C. (2025). Fuchs Endothelial Dystrophy.

[137] Eleiwa T., Elsawy A., Özcan E., Abou Shousha M. (2020). Automated diagnosis and staging of Fuchs’ endothelial cell corneal dystrophy using deep learning. Eye Vis..

[138] Elsawy A., Eleiwa T., Chase C., Ozcan E., Tolba M., Feuer W., Abdel-Mottaleb M., Abou Shousha M. (2021). Multidisease deep learning neural network for the diagnosis of corneal diseases. Am. J. Ophthalmol..

[139] Gu H., Guo Y., Gu L., Wei A., Xie S., Ye Z., Xu J., Zhou X., Lu Y., Liu X. (2020). Deep Learning for Identifying Corneal Diseases from Ocular Surface Slit-Lamp Photographs. Sci. Rep..

[140] Li W., Yang Y., Zhang K., Long E., He L., Zhang L., Zhu Y., Chen C., Liu Z., Wu X. (2020). Dense anatomical annotation of slit lamp images improves the performance of deep learning for the diagnosis of ophthalmic disorders. Nat. Biomed. Eng..

[141] Han S.B., Liu Y.C., Liu C., Mehta J.S. (2024). Applications of Imaging Technologies in Fuchs Endothelial Corneal Dystrophy: A Narrative Literature Review. Bioengineering.

[142] Vigueras-Guillén J.P., van Rooij J., Engel A., Lemij H.G., van Vliet L.J., Vermeer K.A. (2020). Deep Learning for Assessing the Corneal Endothelium from Specular Microscopy Images up to 1 Year after Ultrathin-DSAEK Surgery. Transl. Vis. Sci. Technol..

[143] Laing R.A., Leibowitz H.M., Oak S.S., Chang R., Berrospi A.R., Theodore J. (1981). Endothelial mosaic in Fuchs’ dystrophy. A qualitative evaluation with the specular microscope. Arch. Ophthalmol..

[144] Ong Tone S., Jurkunas U. (2019). Imaging the Corneal Endothelium in Fuchs Corneal Endothelial Dystrophy. Semin. Ophthalmol..

[145] Foo V.H.X., Lim G.Y.S., Liu Y.C., Ong H.S., Wong E., Chan S., Wong J., Mehta J.S., Ting D.S.W., Ang M. (2024). Deep learning for detection of Fuchs endothelial dystrophy from widefield specular microscopy imaging: A pilot study. Eye Vis..

[146] Belda-Para C., Velarde-Rodríguez G., Marichal-Hernández J.G., Velasco-Ocaña M., Trujillo-Sevilla J.M., Alejandre-Alba N., Rodríguez-Ramos J.M. (2024). Fuchs’ Endothelial Corneal Dystrophy evaluation using a high-resolution wavefront sensor. Sci. Rep..

[147] Bitton K., Zéboulon P., Ghazal W., Rizk M., Elahi S., Gatinel D. (2022). Deep Learning Model for the Detection of Corneal Edema Before Descemet Membrane Endothelial Keratoplasty on Optical Coherence Tomography Images. Transl. Vis. Sci. Technol..

[148] Prada A.M., Quintero F., Mendoza K., Galvis V., Tello A., Romero L.A., Marrugo A.G. (2024). Assessing Fuchs Corneal Endothelial Dystrophy Using Artificial Intelligence-Derived Morphometric Parameters from Specular Microscopy Images. Cornea.

[149] Liu S., Kandakji L., Stupnicki A., Sumodhee D., Leucci M.T., Hau S., Balal S., Okonkwo A., Moghul I., Kanda S.P. (2025). Current Applications of Artificial Intelligence for Fuchs Endothelial Corneal Dystrophy: A Systematic Review. Transl. Vis. Sci. Technol..

[150] Yang H.K., Che S.A., Hyon J.Y., Han S.B. (2022). Integration of Artificial Intelligence into the Approach for Diagnosis and Monitoring of Dry Eye Disease. Diagnostics.

[151] Pur D.R., Krance S.H., Pucchio A., Miranda R.N., Felfeli T. (2023). Current Uses of Artificial Intelligence in the Analysis of Biofluid Markers Involved in Corneal and Ocular Surface Diseases: A Systematic Review. Eye.

[152] Mohammadi S.F., Farrokhpour H., Soltani G., Latifi G. (2023). Keratoneuropathy. Ocul. Surf..

[153] Storås A.M., Strümke I., Riegler M.A., Grauslund J., Hammer H.L., Yazidi A., Halvorsen P., Gundersen K.G., Utheim T.P., Jackson C.J. (2022). Artificial Intelligence in Dry Eye Disease. Ocul. Surf..

[154] Persiya J., Sasithradevi A. (2024). Thermal Mapping the Eye: A Critical Review of Advances in Infrared Imaging for Disease Detection. J. Therm. Biol..

[155] Cartes C., López D., Salinas D., Segovia C., Ahumada C., Pérez N., Valenzuela F., Lanza N., López Solís R.O., Perez V.L. (2019). Dry Eye Is Matched by Increased Intrasubject Variability in Tear Osmolarity as Confirmed by Machine Learning Approach. Arch. Soc. Esp. Oftalmol..

[156] Grus F.H., Augustin A.J. (1999). Analysis of Tear Protein Patterns by a Neural Network as a Diagnostical Tool for the Detection of Dry Eyes. Electrophoresis.

[157] Grus F.H., Podust V.N., Bruns K., Lackner K., Fu S., Dalmasso E.A., Wirthlin A., Pfeiffer N. (2005). SELDI-TOF-MS ProteinChip Array Profiling of Tears from Patients with Dry Eye. Investig. Ophthalmol. Vis. Sci..

[158] Yedidya T., Hartley R., Guillon J.-P., Kanagasingam Y. (2007). Automatic Dry Eye Detection. Medical Image Computing and Computer-Assisted Intervention–MICCAI 2007.

[159] Su T.-Y., Liu Z.-Y., Chen D.-Y. (2018). Tear Film Break-Up Time Measurement Using Deep Convolutional Neural Networks for Screening Dry Eye Disease. IEEE Sens. J..

[160] Vyas A.H., Mehta M.A., Kotecha K., Pandya S., Alazab M., Gadekallu T.R. (2024). Tear Film Breakup Time-Based Dry Eye Disease Detection Using Convolutional Neural Network. Neural Comput. Appl..

[161] Su T.-Y., Ting P.-J., Chang S.-W., Chen D.-Y. (2019). Superficial Punctate Keratitis Grading for Dry Eye Screening Using Deep Convolutional Neural Networks. IEEE Sens. J..

[162] Arita R., Yabusaki K., Yamauchi T., Ichihashi T., Morishige N. (2018). Diagnosis of Dry Eye Subtype by Artificial Intelligence Software Based on the Interferometric Fringe Pattern of the Tear Film Obtained with the Kowa DR-1α Instrument. Investig. Ophthalmol. Vis. Sci..

[163] Yabusaki K., Arita R., Yamauchi T. (2019). Automated Classification of Dry Eye Type Analyzing Interference Fringe Color Images of Tear Film Using Machine Learning Techniques. Model. Artif. Intell. Ophthalmol..

[164] Zheng Q., Wang L., Wen H., Ren Y., Huang S., Bai F., Li N., Craig J.P., Tong L., Chen W. (2022). Impact of Incomplete Blinking Analyzed Using a Deep Learning Model with the Keratograph 5M in Dry Eye Disease. Transl. Vis. Sci. Technol..

[165] Zheng Q., Zhang X., Zhang J., Bai F., Huang S., Pu J., Chen W., Wang L. (2022). A Texture-Aware U-Net for Identifying Incomplete Blinking from Eye Videography. Biomed. Signal Process. Control.

[166] Zhang Z.Z., Kuang R.F., Wei Z.Y., Wang L.Y., Su G.Y., Ou Z.H., Liang Q.F. (2022). Detection of the Spontaneous Blinking Pattern of Dry Eye Patients Using the Machine Learning Method. Zhonghua Yan Ke Za Zhi.

[167] Edorh N.A., El Maftouhi A., Djerada Z., Arndt C., Denoyer A. (2022). New Model to Better Diagnose Dry Eye Disease Integrating OCT Corneal Epithelial Mapping. Br. J. Ophthalmol..

[168] Wang M.T.M., Xue A.L., Craig J.P. (2019). Screening Utility of a Rapid Non-Invasive Dry Eye Assessment Algorithm. Cont. Lens Anterior Eye.

[169] Heidari Z., Hashemi H., Sotude D., Ebrahimi-Besheli K., Khabazkhoob M., Soleimani M., Djalilian A.R., Yousefi S. (2024). Applications of Artificial Intelligence in Diagnosis of Dry Eye Disease: A Systematic Review and Meta-Analysis. Cornea.

[170] Aranha dos Santos V., Schmetterer L., Gröschl M., Garhofer G., Schmidl D., Kucera M., Unterhuber A., Hermand J.-P., Werkmeister R.M. (2015). In Vivo Tear Film Thickness Measurement and Tear Film Dynamics Visualization Using Spectral Domain Optical Coherence Tomography. Opt. Express.

[171] Deng X., Tian L., Liu Z., Zhou Y., Jie Y. (2021). A Deep Learning Approach for the Quantification of Lower Tear Meniscus Height. Biomed. Signal Process. Control.

[172] Stegmann H., Aranha dos Santos V., Messner A., Unterhuber A., Schmidl D., Garhöfer G., Schmetterer L., Werkmeister R.M. (2019). Automatic Assessment of Tear Film and Tear Meniscus Parameters in Healthy Subjects Using Ultrahigh-Resolution Optical Coherence Tomography. Biomed. Opt. Express.

[173] Stegmann H., Werkmeister R.M., Pfister M., Garhöfer G., Schmetterer L., dos Santos V.A. (2020). Deep Learning Segmentation for Optical Coherence Tomography Measurements of the Lower Tear Meniscus. Biomed. Opt. Express.

[174] Egrilmez S., Yildirim-Theveny Ş. (2020). Treatment-Resistant Bacterial Keratitis: Challenges and Solutions. Clin. Ophthalmol..

[175] Farrokhpour H., Soleimani M., Cheraqpour K., Masoumi A., Tabatabaei S.A., Shahriari M., Hobaby S., Baharnoor S.M., Chaudhry A., Djalilian A.R. (2023). A Case Series of Infectious Keratitis After Corneal Cross-Linking. J. Refract. Surg..

[176] Soleimani M., Keykhaei M., Tabatabaei S.A., Shahriari M., Farrokhpour H., Ramezani B., Cheraqpour K. (2023). Post Photorefractive Keratectomy (PRK) Infectious Keratitis; Six-Year Experience of a Tertiary Eye Hospital. Eye.

[177] Parikh P.C., Valikodath N.G., Estopinal C.B., Shtein R.M., Sugar A., Niziol L.M., Woodward M.A. (2017). Precision of Epithelial Defect Measurements. Cornea.

[178] Upadhyay M.P., Srinivasan M., Whitcher J.P. (2015). Diagnosing and Managing Microbial Keratitis. Community Eye Health.

[179] Avunduk A.M., Varnell E.D., Kaufman H.E. (2003). The Effect of Roscovitine on Herpetic Keratitis. Exp. Eye Res..

[180] Moshirfar M., Hopping G.C., Vaidyanathan U., Liu H., Somani A.N., Ronquillo Y.C., Hoopes P.C. (2019). Biological Staining and Culturing in Infectious Keratitis: Controversy in Clinical Utility. Med. Hypothesis Discov. Innov. Ophthalmol..

[181] Sarayar R., Lestari Y.D., Setio A.A.A., Sitompul R. (2023). Accuracy of Artificial Intelligence Model for Infectious Keratitis Classification: A Systematic Review and Meta-Analysis. Front. Public Health.

[182] Ong Z.Z., Sadek Y., Qureshi R., Liu S.-H., Li T., Liu X., Takwoingi Y., Sounderajah V., Ashrafian H., Ting D.S.W. (2024). Diagnostic Performance of Deep Learning for Infectious Keratitis: A Systematic Review and Meta-Analysis. EClinicalMedicine.

[183] Soleimani M., Esmaili K., Rahdar A., Aminizadeh M., Cheraqpour K., Tabatabaei S.A., Mirshahi R., Bibak-Bejandi Z., Mohammadi S.F., Koganti R. (2023). From the Diagnosis of Infectious Keratitis to Discriminating Fungal Subtypes; A Deep Learning-Based Study. Sci. Rep..

[184] Wei Z., Wang S., Wang Z., Chen K., Gong L., Li G., Zheng Q., Zhang Q., He Y., Zhang Q. (2023). Development and Multi-Center Validation of Machine Learning Model for Early Detection of Fungal Keratitis. eBioMedicine.

[185] Li Z., Xie H., Wang Z., Li D., Chen K., Zong X., Qiang W., Wen F., Deng Z., Chen L. (2024). Deep Learning for Multi-Type Infectious Keratitis Diagnosis: A Nationwide, Cross-Sectional, Multicenter Study. npj Digit. Med..

[186] Shareef O., Soleimani M., Tu E., Jacobs D.S., Ciolino J.B., Rahdar A., Cheraqpour K., Ashraf M., Habib N.B., Greenfield J. (2024). A Novel Artificial Intelligence Model for Diagnosing Acanthamoeba Keratitis Through Confocal Microscopy. Ocul. Surf..

[187] Ting D.S., Gopal B.P., Deshmukh R., Seitzman G.D., Said D.G., Dua H.S. (2022). Diagnostic Armamentarium of Infectious Keratitis: A Comprehensive Review. Ocul. Surf..

[188] Saini J.S., Jain A.K., Kumar S., Vikal S., Pankaj S., Singh S. (2003). Neural Network Approach to Classify Infective Keratitis. Curr. Eye Res..

[189] Ghosh A.K., Thammasudjarit R., Jongkhajornpong P., Attia J., Thakkinstian A. (2022). Deep Learning for Discrimination Between Fungal Keratitis and Bacterial Keratitis: DeepKeratitis. Cornea.

[190] Hung N., Shih A.K.-Y., Lin C., Kuo M.-T., Hwang Y.-S., Wu W.-C., Kuo C.-F., Kang E.Y.-C., Hsiao C.-H. (2021). Using Slit-Lamp Images for Deep Learning-Based Identification of Bacterial and Fungal Keratitis: Model Development and Validation with Different Convolutional Neural Networks. Diagnostics.

[191] Redd T.K., Prajna N.V., Srinivasan M., Lalitha P., Krishnan T., Rajaraman R., Venugopal A., Acharya N., Seitzman G.D., Lietman T.M. (2022). Image-Based Differentiation of Bacterial and Fungal Keratitis Using Deep Convolutional Neural Networks. Ophthalmol. Sci..

[192] Redd T.K., Santina L.D., Prajna L.V., Lalitha P., Acharya N., Lietman T. (2021). Automated Differentiation of Bacterial from Fungal Keratitis Using Deep Learning. Investig. Ophthalmol. Vis. Sci..

[193] Woodward M.A., Maganti N., Niziol L.M., Amin S., Hou A., Singh K. (2021). Development and Validation of a Natural Language Processing Algorithm to Extract Descriptors of Microbial Keratitis from the Electronic Health Record. Cornea.

[194] Kuo M.T., Hsu B.W., Lin Y.S., Fang P.C., Yu H.J., Chen A., Yu M.S., Tseng V.S. (2021). Comparisons of Deep Learning Algorithms for Diagnosing Bacterial Keratitis via External Eye Photographs. Sci. Rep..

[195] Loo J., Woodward M.A., Prajna V., Kriegel M.F., Pawar M., Khan M., Niziol L.M., Farsiu S. (2021). Open-Source Automatic Biomarker Measurement on Slit-Lamp Photography to Estimate Visual Acuity in Microbial Keratitis. Transl. Vis. Sci. Technol..

[196] Mayya V., Kamath Shevgoor S., Kulkarni U., Hazarika M., Barua P.D., Acharya U.R. (2021). Multi-Scale Convolutional Neural Network for Accurate Corneal Segmentation in Early Detection of Fungal Keratitis. J. Fungi.

[197] Farsiu S., Loo J., Kreigel M.F., Tuohy M., Prajna V., Woodward M.A. (2019). Deep Learning-Based Automatic Segmentation of Stromal Infiltrates and Associated Biomarkers on Slit-Lamp Images of Microbial Keratitis. Investig. Ophthalmol. Vis. Sci..

[198] Ji Q., Jiang Y., Qu L., Yang Q., Zhang H. (2022). An Image Diagnosis Algorithm for Keratitis Based on Deep Learning. Neural Process. Lett..

[199] Kuo M.T., Hsu B.W., Yin Y.K., Fang P.C., Lai H.Y., Chen A., Yu M.S., Tseng V.S. (2020). A Deep Learning Approach in Diagnosing Fungal Keratitis Based on Corneal Photographs. Sci. Rep..

[200] Kriegel M.F., Huang J., Ashfaq H.A., Niziol L.M., Preethi M., Tan H., Tuohy M.M., Patel T.P., Prajna V., Woodward M.A. (2020). Algorithm Variability in Quantification of Epithelial Defect Size in Microbial Keratitis Images. Cornea.

[201] Patel T.P., Prajna N.V., Farsiu S., Valikodath N.G., Niziol L.M., Dudeja L., Kim K.H., Woodward M.A. (2018). Novel Image-Based Analysis for Reduction of Clinician-Dependent Variability in Measurement of the Corneal Ulcer Size. Cornea.

[202] Liu Z., Cao Y., Li Y., Xiao X., Qiu Q., Yang M., Zhao Y., Cui L. (2020). Automatic Diagnosis of Fungal Keratitis Using Data Augmentation and Image Fusion with Deep Convolutional Neural Network. Comput. Methods Programs Biomed..

[203] Wu X., Qiu Q., Liu Z., Zhao Y., Zhang B., Zhang Y., Wu X., Ren J. (2018). Hyphae Detection in Fungal Keratitis Images with Adaptive Robust Binary Pattern. IEEE Access.

[204] Lv J., Zhang K., Chen Q., Chen Q., Huang W., Cui L., Li M., Li J., Chen L., Shen C. (2020). Deep Learning-Based Automated Diagnosis of Fungal Keratitis with In Vivo Confocal Microscopy Images. Ann. Transl. Med..

[205] Hou H., Cao Y., Cui X., Liu Z., Xu H., Wang C., Zhang W., Zhang Y., Fang Y., Geng Y. (2021). Medical Image Management and Analysis System Based on Web for Fungal Keratitis Images. Math. Biosci. Eng..

[206] Koyama A., Miyazaki D., Nakagawa Y., Ayatsuka Y., Miyake H., Ehara F., Sasaki S.-I., Shimizu Y., Inoue Y. (2021). Determination of Probability of Causative Pathogen in Infectious Keratitis Using Deep Learning Algorithm of Slit-Lamp Images. Sci. Rep..

[207] Soleimani M., Rahdar A., Esmaili K., Cheraqpour K., Baharnoori M., Kufta A., Mohammadi S.F., Cheung A., Yousefi S., Djalilian A.R. (2024). AI-Assisted Diagnosis and Subtype Differentiation of Microbial Keratitis: One Step Forward to Mitigate Health Disparities. Investig. Ophthalmol. Vis. Sci..

[208] Vupparaboina K.K., Vedula S.N., Aithu S., Bashar S.B., Challa K., Loomba A., Taneja M., Channapayya S., Richhariya A. (2019). Artificial Intelligence Based Detection of Infectious Keratitis Using Slit-Lamp Images. Investig. Ophthalmol. Vis. Sci..

[209] Xu Y., Kong M., Xie W., Duan R., Fang Z., Lin Y., Zhu Q., Tang S., Wu F., Yao Y.-F. (2021). Deep Sequential Feature Learning in Clinical Image Classification of Infectious Keratitis. Engineering.

[210] Fang Z., Kuang K., Lin Y., Wu F., Yao Y.-F. Concept-Based Explanation for Fine-Grained Images and Its Application in Infectious Keratitis Classification. Proceedings of the 28th ACM International Conference on Multimedia.

[211] Wang L., Chen K., Wen H., Zheng Q., Chen Y., Pu J., Chen W. (2021). Feasibility Assessment of Infectious Keratitis Depicted on Slit-Lamp and Smartphone Photographs Using Deep Learning. Int. J. Med. Inform..

[212] Natarajan R., Matai H.D., Raman S., Kumar S., Ravichandran S., Swaminathan S., Alex John S.R. (2022). Advances in the Diagnosis of Herpes Simplex Stromal Necrotising Keratitis: A Feasibility Study on Deep Learning Approach. Indian J. Ophthalmol..

[213] Tiwari M., Piech C., Baitemirova M., Prajna N.V., Srinivasan M., Lalitha P., Villegas N., Balachandar N., Chua J.T., Redd T. (2022). Differentiation of Active Corneal Infections from Healed Scars Using Deep Learning. Ophthalmology.

[214] Alquran H., Al-Issa Y., Alsalatie M., Mustafa W.A., Qasmieh I.A., Zyout A. (2022). Intelligent Diagnosis and Classification of Keratitis. Diagnostics.

[215] Mansoor H., Tan H.C., Lin M.T., Mehta J.S., Liu Y.C. (2020). Diabetic Corneal Neuropathy. J. Clin. Med..

[216] Vereertbrugghen A., Galletti J.G. (2022). Corneal Nerves and Their Role in Dry Eye Pathophysiology. Exp. Eye Res..

[217] Liu Y.C., Lin M.T., Mehta J.S. (2021). Analysis of Corneal Nerve Plexus in Corneal Confocal Microscopy Images. Neural Regen. Res..

[218] Preston F.G., Meng Y., Burgess J., Ferdousi M., Azmi S., Petropoulos I.N., Kaye S., Malik R.A., Zheng Y., Alam U. (2022). Artificial Intelligence Utilising Corneal Confocal Microscopy for the Diagnosis of Peripheral Neuropathy in Diabetes Mellitus and Prediabetes. Diabetologia.

[219] Salahouddin T., Petropoulos I.N., Ferdousi M., Ponirakis G., Asghar O., Alam U., Kamran S., Mahfoud Z.R., Efron N., Malik R.A. (2021). Artificial Intelligence–Based Classification of Diabetic Peripheral Neuropathy from Corneal Confocal Microscopy Images. Diabetes Care.

[220] Scarpa F., Colonna A., Ruggeri A. (2020). Multiple-Image Deep Learning Analysis for Neuropathy Detection in Corneal Nerve Images. Cornea.

[221] Williams B.M., Borroni D., Liu R., Zhao Y., Zhang J., Lim J., Ma B., Romano V., Qi H., Ferdousi M. (2020). An Artificial Intelligence-Based Deep Learning Algorithm for the Diagnosis of Diabetic Neuropathy Using Corneal Confocal Microscopy: A Development and Validation Study. Diabetologia.

[222] Mou L., Qi H., Liu Y., Zheng Y., Matthew P., Su P., Liu J., Zhang J., Zhao Y. (2022). DeepGrading: Deep Learning Grading of Corneal Nerve Tortuosity. IEEE Trans. Med. Imaging.

[223] Wu X., Liu L., Zhao L., Guo C., Li R., Wang T., Yang X., Xie P., Liu Y., Lin H. (2020). Application of Artificial Intelligence in Anterior Segment Ophthalmic Diseases: Diversity and Standardization. Ann. Transl. Med..

[224] Delgado-Rivera G., Roman-Gonzalez A., Alva-Mantari A., Saldivar-Espinoza B., Zimic M., Barrientos-Porras F., Salguedo-Bohorquez M. (2018). Method for the Automatic Segmentation of the Palpebral Conjunctiva Using Image Processing. Proceedings of the 2018 IEEE International Conference on Automation/XXIII Congress of the Chilean Association of Automatic Control (ICA-ACCA).

[225] Derakhshani R., Saripalle S.K., Doynov P. (2012). Computational Methods for Objective Assessment of Conjunctival Vascularity. Proceedings of the 2012 Annual International Conference of the IEEE Engineering in Medicine and Biology Society.

[226] Jo H.-C., Jeong H., Lee J., Na K.-S., Kim D.-Y. (2021). Quantification of Blood Flow Velocity in the Human Conjunctival Microvessels Using Deep Learning-Based Stabilization Algorithm. Sensors.

[227] Owen C.G., Ellis T.J., Woodward E.G. (2004). A Comparison of Manual and Automated Methods of Measuring Conjunctival Vessel Widths from Photographic and Digital Images. Ophthalmic Physiol. Opt..

[228] Masumoto H., Tabuchi H., Yoneda T., Nakakura S., Ohsugi H., Sumi T., Fukushima A. (2019). Severity Classification of Conjunctival Hyperaemia by Deep Neural Network Ensembles. J. Ophthalmol..

[229] Li X., Xia C., Li X., Wei S., Zhou S., Yu X., Gao J., Cao Y., Zhang H. (2022). Identifying Diabetes from Conjunctival Images Using a Novel Hierarchical Multi-Task Network. Sci. Rep..

[230] Mergen B., Safi T., Nadig M., Bhattrai G., Daas L., Alexandersson J., Seitz B. (2024). Detecting the corneal neovascularisation area using artificial intelligence. Br. J. Ophthalmol..

[231] Mirzayev I., Gündüz A.K., Aydın Ellialtıoğlu P., Gündüz Ö.Ö. (2023). Clinical Applications of Anterior Segment Swept-Source Optical Coherence Tomography: A Systematic Review. Photodiagnosis Photodyn. Ther..

[232] Yoo T.K., Choi J.Y., Kim H.K., Ryu I.H., Kim J.K. (2021). Adopting Low-Shot Deep Learning for the Detection of Conjunctival Melanoma Using Ocular Surface Images. Comput. Methods Programs Biomed..

[233] Ueno Y., Oda M., Yamaguchi T., Fukuoka H., Nejima R., Kitaguchi Y., Miyake M., Akiyama M., Miyata K., Kashiwagi K. (2024). Deep learning model for extensive smartphone-based diagnosis and triage of cataracts and multiple corneal diseases. Br. J. Ophthalmol..

[234] Maehara H., Ueno Y., Yamaguchi T., Kitaguchi Y., Miyazaki D., Nejima R., Inomata T., Kato N., Chikama T.I., Ominato J. (2025). Artificial intelligence support improves diagnosis accuracy in anterior segment eye diseases. Sci. Rep..

[235] Taki Y., Ueno Y., Oda M., Kitaguchi Y., Ibrahim O.M.A., Aketa N., Yamaguchi T. (2024). Analysis of the performance of the CorneAI for iOS in the classification of corneal diseases and cataracts based on journal photographs. Sci. Rep..

[236] Kozma K., Jánki Z.R., Bilicki V., Csutak A., Szalai E. (2025). Artificial intelligence to enhance the diagnosis of ocular surface squamous neoplasia. Sci. Rep..

[237] Nguena M.B., van den Tweel J.G., Makupa W., Hu V.H., Weiss H.A., Gichuhi S., Burton M.J. (2014). Diagnosing ocular surface squamous neoplasia in East Africa: Case-control study of clinical and in vivo confocal microscopy assessment. Ophthalmology.

[238] Li Z., Wang Y., Qiang W., Wu X., Zhang Y., Gu Y., Chen K., Qi D., Xiu L., Sun Y. (2025). A Domain-Specific Pretrained Model for Detecting Malignant and Premalignant Ocular Surface Tumors: A Multicenter Model Development and Evaluation Study. Research.

[239] Wang Y., Yao Q., Kwok J.T., Ni L.M. (2020). Generalizing from a few examples: A survey on few-shot learning. ACM Comput. Surv. (CSUR).

[240] Sarkar P., Tripathy K. (2025). Pterygium.

[241] Chen B., Fang X.W., Wu M.N., Zhu S.J., Zheng B., Liu B.Q., Wu T., Hong X.Q., Wang J.T., Yang W.H. (2023). Artificial intelligence assisted pterygium diagnosis: Current status and perspectives. Int. J. Ophthalmol..

[242] Gan F., Chen W.Y., Liu H., Zhong Y.L. (2022). Application of artificial intelligence models for detecting the pterygium that requires surgical treatment based on anterior segment images. Front. Neurosci..

[243] Zhu S., Fang X., Qian Y., He K., Wu M., Zheng B., Song J. (2022). Pterygium Screening and Lesion Area Segmentation Based on Deep Learning. J. Healthc. Eng..

[244] Wan C., Shao Y., Wang C., Jing J., Yang W. (2022). A Novel System for Measuring Pterygium’s Progress Using Deep Learning. Front. Med..

[245] Hung K.H., Lin C., Roan J., Kuo C.F., Hsiao C.H., Tan H.Y., Chen H.C., Ma D.H., Yeh L.K., Lee O.K. (2022). Application of a Deep Learning System in Pterygium Grading and Further Prediction of Recurrence with Slit Lamp Photographs. Diagnostics.

[246] Liu Y., Xu C., Wang S., Chen Y., Lin X., Guo S., Liu Z., Wang Y., Zhang H., Guo Y. (2024). Accurate detection and grading of pterygium through smartphone by a fusion training model. Br. J. Ophthalmol..

[247] Fang X., Deshmukh M., Chee M.L., Soh Z.D., Teo Z.L., Thakur S., Goh J.H.L., Liu Y.C., Husain R., Mehta J.S. (2022). Deep learning algorithms for automatic detection of pterygium using anterior segment photographs from slit-lamp and hand-held cameras. Br. J. Ophthalmol..

[248] Zheng B., Liu Y., He K., Wu M., Jin L., Jiang Q., Zhu S., Hao X., Wang C., Yang W. (2021). Research on an Intelligent Lightweight-Assisted Pterygium Diagnosis Model Based on Anterior Segment Images. Dis. Markers.

[249] Li Z., Wang Z., Xiu L., Zhang P., Wang W., Wang Y., Chen G., Yang W., Chen W. (2025). Large language model-based multimodal system for detecting and grading ocular surface diseases from smartphone images. Front. Cell Dev. Biol..

[250] Seitzman G.D., Prajna L., Prajna N.V., Sansanayudh W., Satitpitakul V., Laovirojjanakul W., Chen C., Zhong L., Ouimette K., Redd T. (2024). Biomarker Detection and Validation for Corneal Involvement in Patients with Acute Infectious Conjunctivitis. JAMA Ophthalmol..

[251] Delsoz M., Madadi Y., Munir W.M., Tamm B., Mehravaran S., Soleimani M., Djalilian A., Yousefi S. (2024). Performance of ChatGPT in Diagnosis of Corneal Eye Diseases. Cornea.

[252] Hussain Z.S., Delsoz M., Elahi M., Jerkins B., Kanner E., Wright C., Munir W.M., Soleimani M., Djalilian A., Lao P.A. (2025). Performance of DeepSeek, Qwen 2.5 MAX, and ChatGPT Assisting in Diagnosis of Corneal Eye Diseases, Glaucoma, and Neuro-Ophthalmology Diseases Based on Clinical Case Reports. medRxiv.

[253] Jiao C., Rosas E., Asadigandomani H., Delsoz M., Madadi Y., Raja H., Munir W.M., Tamm B., Mehravaran S., Djalilian A.R. (2025). Diagnostic Performance of Publicly Available Large Language Models in Corneal Diseases: A Comparison with Human Specialists. Diagnostics.

[254] Soleimani M., Cheraqpour K., Sadeghi R., Pezeshgi S., Koganti R., Djalilian A.R. (2023). Artificial Intelligence and Infectious Keratitis: Where Are We Now?. Life.

[255] Kim M., Sohn H., Choi S., Kim S. (2021). Requirements for Trustworthy Artifical Intelligence and its Application in Healthcare. Healthc. Inform. Res..

[256] Liu X., Rivera S.C., Moher D., Calvert M.J., Denniston A.K., The SPIRIT-AI and CONSORT-AI Working Group (2020). Reporting Guidelines for Clinical Trials Evaluating Artificial Intelligence Interventions: The CONSORT-AI Extension. npj Digit. Med..

[257] Sounderajah V., Ashrafian H., Golub R.M., Shetty S., De Fauw J., Hooft L., Moons K., Collins G., Moher D., Bossuyt P.M. (2021). Developing a Reporting Guideline for Artificial Intelligence-Centred Diagnostic Test Accuracy Studies: The STARD-AI Protocol. BMJ Open.

[258] Maile H., Li J.P.O., Gore D., Leucci M., Mulholland P., Hau S., Szabo A., Moghul I., Balaskas K., Fujinami K. (2021). Machine Learning Algorithms to Detect Subclinical Keratoconus: Systematic Review. JMIR Med. Inform..

[259] Guni A., Sounderajah V., Whiting P., Bossuyt P., Darzi A., Ashrafian H. (2024). Revised Tool for the Quality Assessment of Diagnostic Accuracy Studies Using AI (QUADAS-AI): Protocol for a Qualitative Study. JMIR Res. Protoc..

[260] Kazemzadeh K. (2025). Artificial intelligence in ophthalmology: Opportunities, challenges, and ethical considerations. Med. Hypothesis Discov. Innov. Ophthalmol. J..

[261] Li Q., Tan J., Xie H., Zhang X., Dai Q., Li Z., Yan L.L., Chen W. (2024). Evaluating the accuracy of the Ophthalmologist Robot for multiple blindness-causing eye diseases: A multicentre, prospective study protocol. BMJ Open.

[262] Young J.A., Chang C.W., Scales C.W., Menon S.V., Holy C.E., Blackie C.A. (2024). Machine Learning Methods Using Artificial Intelligence Deployed on Electronic Health Record Data for Identification and Referral of At-Risk Patients from Primary Care Physicians to Eye Care Specialists: Retrospective, Case-Controlled Study. JMIR AI.

